# Learning to utilize internal protein 3D nanoenvironment descriptors in predicting CRISPR–Cas9 off-target activity

**DOI:** 10.1093/nargab/lqaf054

**Published:** 2025-05-21

**Authors:** Jeffrey Kelvin Mak, Artemi Bendandi, José Augusto Salim, Ivan Mazoni, Fabio Rogerio de Moraes, Luiz Borro, Florian Störtz, Walter Rocchia, Goran Neshich, Peter Minary

**Affiliations:** Department of Computer Science, University of Oxford, Parks Road, Oxford OX1 3QD, United Kingdom; CONCEPT Lab, Istituto Italiano di Tecnologia, Via Melen – 83, B Block, 16152Genova, Italy; Department of Plant Biology, Institute of Biology, University of Campinas – UNICAMP, SP, 13083-872, Brazil; Computational Biology Research Group, Embrapa Digital Agriculture, Campinas, SP, 13083-886, Brazil; Physics Department, Institute of Biosciences, Languages, and Exact Sciences (IBILCE), São Paulo State University (Unesp), São José do Rio Preto, SP, 15054-000, Brazil; beOn Claro, São Paulo, SP, 04709-110, Brazil; Department of Computer Science, University of Oxford, Parks Road, Oxford OX1 3QD, United Kingdom; CONCEPT Lab, Istituto Italiano di Tecnologia, Via Melen – 83, B Block, 16152Genova, Italy; Computational Biology Research Group, Embrapa Digital Agriculture, Campinas, SP, 13083-886, Brazil; Department of Computer Science, University of Oxford, Parks Road, Oxford OX1 3QD, United Kingdom

## Abstract

Despite advances in determining the factors influencing cleavage activity of a CRISPR–Cas9 single guide RNA (sgRNA) at an (off-)target DNA sequence, a comprehensive assessment of pertinent physico-chemical/structural descriptors is missing. In particular, studies have not yet directly exploited the information-rich internal protein 3D nanoenvironment of the sgRNA–(off-)target strand DNA pair, which we obtain by harvesting 634 980 residue-level features for CRISPR–Cas9 complexes. As a proof-of-concept study, we simulated the internal protein 3D nanoenvironment for all experimentally available single-base protospacer-adjacent motif-distal mutations for a given sgRNA–target strand pair. By determining the most relevant residue-level features for CRISPR–Cas9 off-target cleavage activity, we developed STING_CRISPR, a machine learning model delivering accurate predictive performance of off-target cleavage activity for the type of single-base mutations considered in this study. By interpreting STING_CRISPR, we identified four important Cas9 residue spatial hotspots and associated structural/physico-chemical descriptor classes influencing CRISPR–Cas9 (off-)target cleavage activity for the sgRNA–target strand pairs covered in this study.

## Graphical Abstract

**Graphical Abstract:**
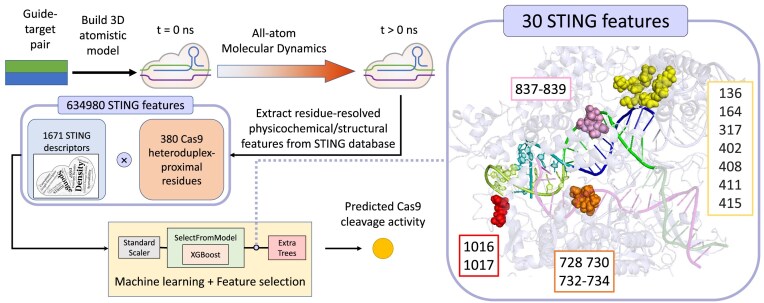

## Introduction

CRISPR–Cas9 is a programmable RNA-guided endonuclease which originates from adaptive bacterial defense systems [1–3]. The CRISPR–Cas9 genome editor is composed of a Cas9 nuclease and a single guide RNA (sgRNA) [4]. Cas9, which stands for CRISPR-associated protein 9, is a bi-lobed enzyme, in which the sgRNA is placed between the alpha-helical lobe (called REC), which mediates nucleic acid binding, and the nuclease lobe (containing the RuvC and HNH domains), which mediates DNA cleavage. Cas9 genome editing involves three stages. First, the protospacer-adjacent motif (PAM)-interacting domain (PI) of Cas9 recognizes the PAM (5′-NGG in the case of SpCas9). R-loop formation then takes place, consisting of the unwinding of the targeted sequence (on-target)’s double-stranded DNA (dsDNA) and sgRNA–target strand DNA (sgRNA–tsDNA) heteroduplex formation via complementary base pairing between the sgRNA’s spacer sequence and target site DNA’s target strand [2]. Finally, the Cas9 enzyme cleaves the DNA in a specific spot, typically 3- to 4-bp upstream of the PAM [5].

The Cas9 nuclease may also cleave off-targets, i.e. genomic DNA sequences containing mismatches with respect to the sgRNA, which results in undesired cleavage. The possibility of off-target cleavage depends on the number of mismatches, their position, and the type of mismatch [6, 7]. For example, a PAM-distal 4-bp mismatch can trap the catalytic HNH domain in an inactive conformation, but mismatches at PAM-proximal positions preserve the shape of the RNA:DNA hybrid [8]. Accurate identification of all potential off-target sites and evaluation of their activities have been the goals of various computational tools [9–17].

Machine learning (ML) has been instrumental in building the most widely used and efficient (as evaluated by prediction accuracy) models for on/off-target activity prediction [9, 10, 18]. These models require the careful selection of relevant features related to the activity of a given sgRNA at a potential (on/off) target site. Some of the most widely used observed features originate from pioneering work on optimized sgRNA design [13, 19] and include (but are not limited to) dinucleotide and single-nucleotide identities at each position of the sgRNA, position independent nucleotide counts, the location of the sgRNA within the gene, the GC count of the sgRNA, as well as thermodynamic features. These features were first used to feed ‘traditional’ predictive ML methods, e.g. regularized linear regression, support vector machines [20], random forest [21], and gradient-boosted regression trees [22–25], just to mention a few. Deep learning (DL)-based off-target prediction models [9, 10] were also proposed. Deep neural networks [26] have the advantage of high prediction accuracy but make model interpretation more challenging and need a large amount of training data.

Current state-of-the-art DL approaches [27–30] for off-target activity prediction complement the sequence features with a diverse set of physically inspired scores such as approximate energy terms [16] for sgRNA–tsDNA hybridization and epigenetic features essential for off-target activity [27], but have not yet directly exploited knowledge based on the information-rich internal 3D local structure (protein) environment surrounding the sgRNA–tsDNA sequence pair, which has been investigated in various experimental studies [7, 31]. This work aims to make the first step towards filling this gap and paves the way for a new generation of models that are rooted in the paradigms of rational design, interpretability, and explainability, and therefore aspires to deliver a deeper insight into the mechanistic factors that underlie (off-)target cleavage activity in CRISPR–Cas9 gene editing.

Atomistic molecular dynamics (MD) has been used to characterize the functioning of the CRISPR–Cas9 systems, providing trajectories and therefore a series of conformations for systems with distinct base pair mismatches at PAM-distal sites of the sgRNA–tsDNA heteroduplex. Here, we found that the modulation of cleavage activity induced by a base pair mismatch at PAM-distal sites is captured by the internal protein 3D nanoenvironment of the sgRNA–tsDNA pair, hereon referred to as ‘nanoenvironment’. In particular, we studied the role of different descriptors and amino acid residues in order to build and train an ML model—named STING_CRISPR—for CRISPR–Cas9 off-target activity prediction of all possible single PAM-distal mismatches of the target of a given sgRNA. This novel approach led to high accuracy (measured in terms of Spearman and Pearson correlations) of experimental off-target activity prediction for sgRNA–tsDNA pairs with single PAM-distal mismatches of a given sgRNA (further referred to as studied sgRNA–tsDNA pairs). However, our presented model unlike established models is not yet capable of predicting cleavage activity for any sgRNA–tsDNA pair. Therefore, this study does not aim for the development of a general CRISPR–Cas9 off-target activity prediction model but the presentation of a proof-of-concept investigation of utilizing the internal protein 3D nanoenvironment for CRISPR–Cas9 off-target activity prediction. Scikit-learn’s SelectFromModel feature selection step [32] in the trained ML pipeline revealed that density, side chain orientation (SCO), accessibility, weighted contact number entropy density, electrostatic potential, sponge, cross presence order, contact energy density, graph descriptor, and solvation, measured at 23 Cas9 residues are of fundamental importance for off-target cleavage activity prediction for the studied sgRNA–tsDNA pairs (see the Supplementary material for the specific definition of each descriptor). Our results lay the foundations for a new type of interpretable ML models capable of predicting CRISPR–Cas9 off-target activity.

## Materials and methods

**Figure 1.**
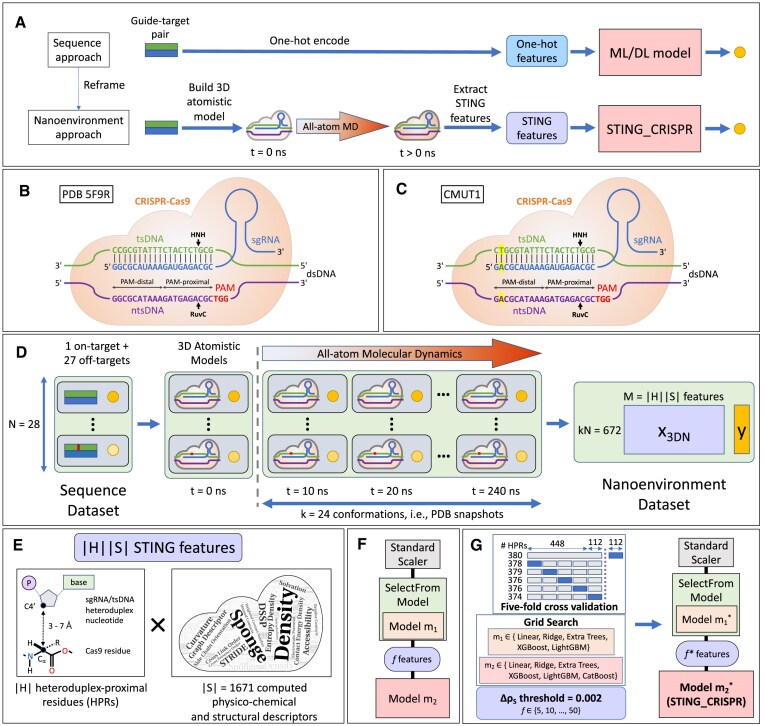
Schematic summary for obtaining STING_CRISPR, our ML model predicting CRISPR–Cas9 cleavage activity for the studied sgRNA–tsDNA pairs. (**A**) Comparison between the purely sequence-based and the nanoenvironment-based approaches for CRISPR–Cas9 cleavage activity prediction by using an ML/DL model and STING_CRISPR, respectively. Catalytically active CRISPR–Cas9 complexes with sgRNA (middle blue strand) and dsDNA (target: top green strand, non-target: bottom purple strand) in PDB 5F9R crystal structure (**B**) and CMUT1 (**C**). Yellow highlights at position +19 show the nucleotides mutated in CMUT1 compared with 5F9R. The leftmost PAM-distal base pair is +20, and the rightmost PAM-proximal base pair is +1. The 20 sgRNA–tsDNA base pairs (vertical black lines) form the heteroduplex. Both DNA strands are cleaved (black arrows) by the HNH and RuvC domains of CRISPR–Cas9, respectively. (**D**) The three-step data pipeline for generating the residue-resolved nanoenvironment dataset from the guide–target dataset. (**E**) The nanoenvironment dataset contains |*H*||*S*| residue-resolved STING features, namely |*S*| = 1671 STING, i.e. physico-chemical and structural, descriptors, each one evaluated at |*H*| sgRNA–tsDNA heteroduplex-proximal residues (HPRs). (**F**) Our ML pipeline which predicts CRISPR–Cas9 cleavage activity, with hyperparameters *m*_1_, *m*_2_, and *f*. (**G**) Grid search with five-fold cross-validation to optimize models *m*_1_ and *m*_2_, followed by feature set size reduction via thresholding of Spearman correlation change (Δρ_S_) to find f*, resulting in a pipeline with hyperparameters $m_1^*$, $m_2^*$, and f*. Shown on the top left, the number of HPRs |*H*| vary for different train-test splits (with the training and test partitions in grey and blue, respectively) during performance evaluation and five-fold cross-validation.

The importance of accurately predicting the (off-)target cleavage activities of the CRISPR–Cas9 gene editing system fueled the application of ML/DL models developed for this prediction task. Most approaches presented in the literature [9, 10, 18] build on labeled datapoints that contain the sgRNA (or guide) and the (off-)target DNA sequences *s*_g_, *s*_t_ together with an experimentally derived cleavage activity label, *a*. A given dataset with *N* such datapoints, $\bigl \lbrace \bigl (s_{\rm g}^{(i)}, s_{\rm t}^{(i)}, a_i\bigl )\bigl \rbrace _{i=1}^{N}$ can be partitioned into training, validation, and test sets so that models for (off-)target cleavage activity prediction can be constructed. Recent years have witnessed the development of a variety of customized models [9, 10, 18] that use distinct DL architectures and/or encoding of the guide and target sequence pair. While all these approaches bring distinct technical contributions, they all aim to learn the following function:


(1) \begin{eqnarray*}
f_{a}: S_{\rm g} \times S_{\rm t} \rightarrow \mathbb {R}, (s_{\rm g},s_{\rm t}) \mapsto f_{a}(s_{\rm g},s_{\rm t}),
\end{eqnarray*}

where *S*_g_ and *S*_t_ are the sets of all guide and target sequences, respectively, and *f*_*a*_ is the functional map from a pair of said sequences to activity (see sequence approach in Fig. 1A). The availability of comprehensive datasets [11] is fundamental for producing models capable of accurately predicting activities associated with unseen guide and target sequences. Methods aiming to learn the function shown by equation (1) have to use data restricted to a particular Cas enzyme (most commonly SpCas9) and by construction they are incapable of predicting changes in activity caused by amino acid residue mutations in the Cas enzyme. The availability of models that predict cleavage activity based on local physical and chemical properties which can be traced back to the amino acid composition of the Cas enzyme would be of utmost importance as they would catalyse the development of bioengineered Cas enzymes with maximal specificity and efficiency.

To meet this objective, we reframe the learning task to that of deciphering the relationship between target cleavage activity and the 3D nanoenvironment—a collection of features characterizing the sgRNA–dsDNA–Cas9 complex, namely the Cas enzyme and the environment encapsulating the guide/target pair in the CRISPR–Cas9 complex (see Fig. 1A). The 3D nanoenvironment is represented by a vector in $\mathbb {R}^M$ where *M* is a suitable integer we determine for the system. A vector can be derived based on a conformation of the sgRNA–dsDNA–Cas9 complex with zero or more nucleotide mutations in the sgRNA, tsDNA, and/or non-target strand DNA (ntsDNA). We can obtain a vector for a given sgRNA–dsDNA–Cas9 complex via the following two steps: (i) construct a 3D atomistic model of the said complex, and (ii) obtain *M* residue-resolved features characterizing the structural and physico-chemical properties of the complex by calculating the STING features for its atomistic model (see nanoenvironment approach in Fig. 1A). We realize that the same sgRNA–dsDNA–Cas9 complex may assume various distinct conformations each giving rise to a potentially distinct 3D nanoenvironment. Therefore as the conformation of the sgRNA–dsDNA–Cas9 complex may dynamically change so does the 3D nanoenvironment calculated from it. To account for having multiple conformations representing sgRNA–dsDNA–Cas9 complexes, we performed MD calculations to generate dynamical trajectories based on the atomistic model for each sgRNA–dsDNA–Cas9 complex and obtain the *M* features (representing the 3D nanoenvironment) for each of the *k* model conformations (snapshots) we sample from each MD trajectory (see Fig. 1D). The implementation details of these steps are discussed in the sections ‘MD of the CRISPR–Cas9 complex with guide–target pair’ and ‘STING descriptors for CRISPR–Cas9 complex with a guide–target pair’. Given that in this study we consider *N* distinct sgRNA–dsDNA–Cas9 complexes (based on the distinct sgRNA–dsDNA pairs) and *k* conformations for each complex (obtained from the corresponding MD trajectories), we altogether consider *kN* conformations. Obtaining the 3D nanoenvironment for each of these conformations results in *kN* distinct 3D nanoenvironments. Furthermore, we may label each 3D nanoenvironment with the experimental cleavage activity of the corresponding sgRNA–dsDNA pair. Thus, we can obtain a labeled dataset $D = \bigl \lbrace \bigl (x_i, a_i\bigl )\bigl \rbrace _{i=1}^{kN}$, where $x_i \in \mathbb {R}^M$ and $a_i \in \mathbb {R}$ (see nanoenvironment dataset in Fig. 1D). Having this labeled dataset enables us learn the relationship between 3D nanoenvironment and cleavage activity. Therefore, formally we aim to learn the following function:


(2) \begin{eqnarray*}
\bar{f}_a: \Omega _{{\rm 3DN}} \rightarrow \mathbb {R}, x \mapsto \bar{f}_a(x),
\end{eqnarray*}

where $\Omega _{{\rm 3DN}} \subset \mathbb {R}^{M}$ and $\bar{f}_a(x)$ is a functional map that takes a vector in $\mathbb {R}^M$ as input and then return a cleavage activity. The dimension *M* of the vector *x*_*i*_ can depend on the degree of detail we choose for describing the 3D nanoenvironment.

Having the dataset $\bigl \lbrace \bigl (x_i, a_i\bigl )\bigl \rbrace _{i=1}^{kN}$ enables us to train a regression model to decipher the relationship between experimental cleavage activity and 3D nanoenvironment. Details on the regression model with feature selection are discussed in the section ‘ML models for CRISPR–Cas9 cleavage activity prediction from STING descriptors’.

### MD of the CRISPR–Cas9 complex with guide–target pair

MD simulations were performed using GROMACS version 2020.2 [33], using bsc1 and AMBER force fields for nucleic acids and protein atoms, respectively. For water molecules, the TIP3P model was used. Protonation states of titratable residues were estimated using the pypKa server [34]. Before the production runs, structures were subjected to NVT equilibration for 400 ps using the modified Berendsen thermostat, and to 1 ns of NPT equilibration using the Parinello–Rahman barostat.

#### Targeted MD

We chose as a reference structure for the enzyme and RNA sequence the crystal structure of the catalytically active *Streptococcus pyogenes* Cas9, primed for target DNA cleavage, in complex with single-stranded guide RNA and dsDNA (both target and non-target strands). The PDB code of this structure is 5F9R, released in 2016. 5F9R has become the most commonly used reference in the literature in recent years. Interestingly, in 2019 the 6O0Y structure was released [35]. Obtained via cryo-electron microscopy (cryo-EM), 6O0Y shows the conformation of the two key domains RuvC and HNH in the catalytically competent state. 5F9R and 6O0Y have the same sgRNA sequence. However, 6O0Y is lacking some key residues and atoms. Therefore, we decided to use the structural information contained in 6O0Y to adapt the conformation of the more complete 5F9R structure. To do this, we performed all-atom explicit solvent targeted MD (TMD) using PLUMED [36–38] as a plugin of GROMACS, in order to bring the RuvC and HNH domains of 5F9R to their catalytically active conformation, mutated from the 6O0Y structure. More specifically, the bias was applied to the heavy atoms of the two protein domains. The collective variable used was the root mean square deviation (RMSD), using a moving restraint with κ going from 0 to 10^5^ in 1.5 × 10^8^ steps.

#### Reference choice and mutants generation

In order to identify our reference sequence for the analysis, we applied the following requirements by filtering the crisprSQL database [11]: having an sgRNA sequence identical or as close as possible to that of the structural reference; having a sufficient number of singly mutated entries in the PAM-distal region of the target DNA strand; and the candidate sequence and the mutated entries must have experimental off-target cleavage activity data. We therefore selected an entry which differs only in one position (RNA base number 2) with respect to the 5F9R and 6O0Y structures and fulfils the other mentioned requirements. For this entry, 28 singly mutated and experimentally annotated other entries were found in the database. We then first mutated base 2 of RNA to adenine and base 29 of the tsDNA to thymine in our reference structure in order to make it identical to the reference sequence, and called it CMUT1 (see Fig. 1B and C). Then we generated the same 28 mutations that were also present in the database on the DNA target strand of CMUT1. Base mutations were done using the software UCSF CHIMERA [39]. Each of them presents only a single mutation, located in the target DNA strand with respect to our reference. A table of the mutations, with associated nomenclature, can be found in Supplementary Table S1.

#### Unbiased MD

We performed 1 µs of all-atom explicit solvent unbiased MD on the output of the TMD, in order to evaluate the dynamics of the structure and to obtain a reference against which to compare further simulations. We also performed 250 ns of all-atom, explicit solvent, unbiased MD on the TMD’s output for each mutant.

#### Electrostatic calculations

We performed electrostatic calculations using the Poisson–Boltzmann equation finite differences solver DelPhi [40]. We calculated the electrostatic energies (partitioned in Coulombic and reaction-field contributions) and the electrostatic potential at the atom centres in order to characterize the local potential on snapshots extracted every 10 ns from the MD trajectory of each mutation. Atomic radii and charges were taken from the AMBER force field [41].

#### RMSD calculations

To evaluate the dynamics of the system, we calculated the RMSD of the following residues for each mutation along the MD trajectory:

- Protein residues (136, 164, 268, 317, 402, 408, 411, 415, 728, 730, 732, 733, 734, 837, 838, 839, 908, 919, 1010, 1016, 1017, 1025, and 1122). These residues were selected based on the following two criteria: they either emerged as significant residues from our ML analysis (see the section ‘Characterization of the heteroduplex-proximal CRISPR–Cas9 internal protein nanoenvironment’ under the section ‘ML models for CRISPR–Cas9 cleavage activity prediction from STING descriptors’).
- RNA and DNA bases belonging to the heteroduplex: chains B and C.

We calculated the RMSD of the nucleic backbone and of the following atoms: C4 and N9 (purines); C6 and N1 (pyrimidines). We also calculated the RMSD of the phosphorus atoms and the N9 and N1 atoms (respectively). This analysis was performed using the MDAnalysis Python package [42].

#### Structure naming scheme

Structures were given a four-character identifier, similar to a PDB code. The first character is a letter, identifying the starting structure for the mutation. We had two kinds of starting structures, the result of our TMD (C for Cryo) and 5F9R (X for X-ray). The second character is either a number from 0 to 9 or a letter from A to Z, and identifies the specific mutation in numerical order from 0 to 9 for the first 10 mutants and then letters in alphabetical order for the remaining ones. The third and fourth character are digits which indicate the snapshot number.

### STING descriptors for CRISPR–Cas9 complex with a guide–target pair

In this study, we consider 60 physico-chemical/structural descriptor classes available from the STING platform database (see Table 1). This translates to 1671 descriptors being organized into the relational database STING_RDB_2_CRISPR, namely one that allows the simultaneous analysis of multiple structures. A concise outline of the 1671 descriptors is included in the section 1 of the Supplementary material, and full descriptions for all STING parameters/descriptors published previously on STING’s web-server site can be found at http://www.cbi.cnptia.embrapa.br/SMS/STINGm/help/MegaHelp_JPD.html and in several papers [43–47]. In this work, we first adopted and then used STING SDL (sting descriptor library), an in-house program able to calculate the descriptors in all possible variants (meaning, using all values for variables employed into formulas that calculate each one of STING descriptors) and applying batch calculations on the sgRNA–dsDNA–Cas9 complexes analysed in MD simulations.

**Table 1.** List of 60 STING descriptor classes (bolded) considered in this study for characterizing the internal protein 3D nanoenvironment of CRISPR–Cas9’s sgRNA–tsDNA heteroduplex

Parent descriptor classes	Associated neighbour descriptor classes
**Accessibility**	
Cross link order (CLO)	**CLO-GN, CLO-SW, CLO-WNA, CLO-VD**
Cross presence order (CPO)	**CPO-GN, CPO-SW, CPO-WNA, CPO-VD**
Curvature (Curv)	**Curv-GN, Curv-SW, Curv-WNA, Curv-VD**
Density	**Density-GN, Density-SW, Density-WNA, Density-VD**
**DSSP**	
Contact energy density (CED)	**CED-GN, CED-SW, CED-WNA, CED-VD**
Electrostatic potential (EP)	**EP-GN, EP-SW, EP-WNA, EP-VD**
Entropy density (ED)	**ED-GN, ED-SW, ED-WNA, ED-VD**
Graph descriptor (GD)	**GD-GN, GD-SW, GD-WNA, GD-VD**
**Hydrophobicity**	
Residue contacts (RC)	**RC-GN, RC-SW, RC-WNA, RC-VD**
Side chain orientation (SCO)	**SCO-GN, SCO-SW, SCO-WNA, SCO-VD**
Solvation (Solv)	**Solv-GN, Solv-SW, Solv-WNA, Solv-VD**
Sponge	**Sponge-GN, Sponge-SW, Sponge-WNA, Sponge-VD**
**STRIDE**	
Unused contacts (UC)	**UC-GN, UC-SW, UC-WNA, UC-VD**
Weighted contact number	**WCN-GN, WCN-SW, WCN-WNA, WCN-VD**

Originating from 18 parent descriptor classes (left column), the 60 descriptor classes consist of 4 parent descriptor classes (bolded, left column) and 56 neighbour descriptor classes (bolded, right column) arising from the application of graph neighbours (GN), sliding window (SW), weighted neighbour average (WNA), and Voronoi diagram (VD) aggregations to 14 other parent descriptor classes (unbolded, left column).

These descriptors were calculated in correspondence of all atoms and in the presence of DNA or RNA bases at distances of 3, 5, and 12 Å from the phosphates for each snapshot. Atom presence lists were generated using custom Python scripts, in which atomic coordinates were parsed using Biopython [48].

### ML for CRISPR–Cas9 cleavage activity prediction from STING descriptors

#### Dataset

Fig. 1 outlines our approach for building STING_CRISPR. Namely, by generating atomistic MD trajectories and computing residue-resolved STING feature values for the atomistic model conformations, we are able to convert our labelled sequence dataset containing 1 on-target and 27 single-mismatch off-target sites into a labelled nanoenvironment dataset of size 672 (Fig. 1D, see raw data in Supplementary Fig. S1 and Supplementary Table S1).

We hypothesize that the internal protein 3D nanoenvironment proximal to Cas9’s sgRNA–tsDNA heteroduplex in the catalytically active conformation is indicative of CRISPR–Cas9 cleavage activity. Moreover, a STING descriptor’s value varies across Cas9 residues, as the value of a physico-chemical or structural property is always tied to a local region/district, i.e. a Cas9 residue in our case. Taking these two ideas into account, we formulate *x*_*i*_ as a vector of length *M* = |*H*||*S*| (see Fig. 1E), where

- *H* denotes the set of HPRs whose α-carbon atoms are 3 to 7 Å away from the C4’ atoms of any sgRNA–tsDNA heteroduplex nucleotide in at least one of the training PDB snapshots, and
- *S* denotes the set of 1671 STING neighbour descriptors available in STING_RDB_2_CRISPR (see Table 1 and Supplementary Table S2) [49–51].

In other words, *x*_*i*_ is a vector containing features (or independent variables) defined by a given STING descriptor at a particular heteroduplex-proximal Cas9 residue, i.e. a STING descriptor–Cas9 residue pair. When computing *H*, we limit distance calculations to training PDB snapshots to avoid data leakage when training ML models.

For STING_CRISPR, we compute 1671 physico-chemical and structural descriptors on 380 HPRs, which resulted in a nanoenvironment dataset with 634 980 STING features (Fig. 1E, see a breakdown of the feature counts in Supplementary Table S2), where the feature values are aggregated over residues within a local neighbourhood as defined by four different aggregation methods available in the STING_RDB_2_CRISPR database—GN, SW, WNA, and VD. See the section ‘Training’ for an explanation on how 380 HPRs were obtained for STING_CRISPR.

#### Exploratory analysis with heteroduplex base pair distances

For each PDB snapshot in the dataset, we compute the Euclidean distance between the two C4’ atoms in each of the 19 PAM-proximal base pairs. We then use a heatmap for each off-target trajectory in order to visualize the heteroduplex base pair distances across all snapshots within each off-target trajectory. As a measure of heteroduplex plasticity, we sum all Euclidean distances across the 19 base pairs over all snapshots for all on- and off-target trajectories. To examine the relationship between this measure and CRISPR–Cas9 cleavage activity, we create violin plots for four groups of sums, namely the sums corresponding to the on-target trajectory, trajectories with low (<0.01) activity, trajectories with medium (0.01–0.1) activity, and trajectories with high (>0.1) activity. We also create a scatter plot between the sums and cleavage activities.

#### ML model

To decipher the relationship between experimental cleavage activity and the 3D nanoenvironment, we build an interpretable scikit-learn [32] ML pipeline (see Fig. 1F) consisting of the following three steps:

- StandardScaler. This scales features to zero mean and unit variance.
- SelectFromModel utilizes base model *m*_1_ and all |*H*||*S*| features to train *m*_1_ and SelectFromModel selects the *f* ≪ |*H*||*S*| most important features from the |*H*||*S*| available features.
- ML model *m*_2_ with *f* input features.

Notably, we embed a feature selection step, i.e. SelectFromModel, into our pipeline, in order to combat the curse of dimensionality [52], and to ensure that *f* is significantly smaller than the training dataset size in our final interpretable ML model.

#### Training

Summarized in Fig. 1G, the training procedure for obtaining STING_CRISPR is as follows. To prepare the data partitions, we first split the dataset into training and test partitions of size 560 and 112 by holding out the last 4 PDB snapshots in from all MD trajectories for testing. Such a split ensures that points in the training and test datasets are distributed similarly. We then randomly split the training partition into five folds for five-fold cross-validation, resulting in five sets of training and validation datasets of size 448 and 112, respectively. Given that models *m*_1_ and *m*_2_ are tunable hyperparameters in the ML pipeline, we first perform grid search with five-fold cross-validation to optimize hyperparameters *m*_1_ and *m*_2_ in the ML pipeline. Specifically, we use grid search to consider the following 5 · 6 · 10 = 300 ML pipelines by using the following hyperparameter ranges:

- model *m*_1_ being either a linear, ridge, XGBoost [23], extra trees [53], or LightGBM [24] model with default hyperparameters (all together five possibilities);
- model *m*_2_ being either a linear, ridge, XGBoost, extra trees, LightGBM, or CatBoost [25] model with default hyperparameters (all together 10 possibilities); and
- number of possible feature size selections |*F*| = 10, where *F* = {5, 10, ..., 50}. We choose such an *F* not only because all elements *f* ∈ *F* satisfy *f* ≪ |*H*||*S*|, but also because we hypothesize that many of the STING features are correlated, meaning that the optimal feature set size is approximately $\sqrt{448} \approx 21.2$ given a training data size of 448 during five-fold cross-validation [54].

We then measure the mean five-fold Spearman correlation validation performance ρ_S_(*m*_1_, *m*_2_, *f*) of each combination (*m*_1_, *m*_2_, *f*), and subsequently find the model pair $(m_1^*, m_2^*)$ with the highest validation performance when averaging the mean Spearman correlation across the 10 possible feature size selections. Once the model pair is found, we pick the smallest feature set size f* such that increasing the selected feature set size by 5 improves the resulting mean five-fold Spearman correlation validation performance by no more than Δρ_S_ = 2 × 10^−3^ (a hyperparameter which thresholds Spearman improvement). Using the hyperparameter configuration $(m_1^*, m_2^*, f^*)$, we then train a single ML pipeline on all 560 points from the training partition. Once trained, we extract *m*_2_ from the pipeline to obtain STING_CRISPR.

Since the HPR set *H* is dependent on the training PDB snapshots, it is worth noting that the training procedure uses six HPR sets, namely one for each fold in five-fold cross-validation, and one extra when training the final model (see top left of Fig. 1G for the HPR set sizes, and the section ‘Heteroduplex-proximal residues’ in the Supplementary material for the specific residues in the six HPR sets). In practice, HPR set sizes of 378, 379, 376, 376, and 374 are obtained for the training sets used in folds 1–5 during five-fold cross-validation, respectively. When performing residue-heteroduplex nucleotide distance calculations on the entire training partition of the nanoenvironment dataset, we identify 380 sgRNA–tsDNA HPRs.

**Figure 2.**
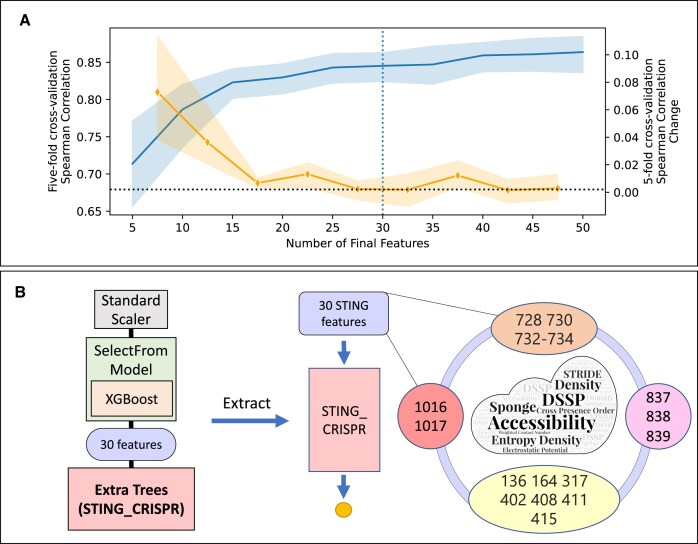
STING_CRISPR is an extra trees model with 30 STING features at 4 residue clusters. (**A**) Hyperparameter tuning of input feature set size in the ML pipeline after grid search with five-fold cross-validation. The solid rising blue line (left *y*-axis) indicates average five-fold Spearman test correlation, and the solid falling orange line (right *y*-axis) indicates average change in the average five-fold test Spearman correlation when increasing the input feature set size in increments of 5. Black dotted horizontal line indicates the Spearman change threshold Δρ_S_ = 0.002, and the blue dotted vertical line indicates the final input feature size selected. (**B**) Extraction of the second ML model (left bottom red box with bolded text) from the hyperparameter-optimized ML pipeline with *m*_1_ = XGBoost, *m*_2_ = extra trees, and *f* = 30 features yields STING_CRISPR, an extra trees model with 30 STING features. Among the 30 STING features, 17 of them form 4 residue clusters (defined below) found to be important in cleavage activity prediction for the studied sgRNA–tsDNA pairs.

In practice, this grid search strategy (see the bullet points above) yields the XGBoost–extra trees combination, which has a mean five-fold cross-validation Spearman correlation of 0.826 when averaged across 10 XGBoost–extra trees pipelines with 5–50 features (Fig. 1G). Illustrated in Fig. 2A, subsequent application of the Spearman correlation change threshold with value 0.002 on the XGBoost–extra trees combination results in a pipeline with 30 features (see Supplementary Table S3 for the list of 30 features). By setting such a threshold, we are able to minimize the feature set size without sacrificing model performance. Together with SelectFromModel, the threshold drastically reduces the ML pipeline’s feature set size from 634 980 to 30 features. By extracting the cleavage activity model from the ML pipeline (see Fig. 2B), we obtain an extra trees model with 30 features, which we name as STING_CRISPR (Fig. 2, red vertical box) in this study. In summary, from the nanoenvironment dataset, the training procedure produced an ML pipeline which feeds the top 30 most important STING features selected from the trained XGBoost surrogate model into an extra trees model for Cas9 activity prediction.

#### Evaluation

We record STING_CRISPR’s performance on the test dataset for the following metrics: Spearman correlation, Pearson correlation, mean squared error, and mean absolute error. Using test data, we also use bar plots to visualize the mean and standard deviation of the square errors between predicted and actual cleavage activities for the on-target interface, PAM-distal mismatch positions, and mismatch interface types.

#### Model interpretation

Our framework for interpreting STING_CRISPR is founded on feature counts and SHapley Additive exPlanations (SHAP) [55] (a summary of the theory behind SHAP can be found in the Supplementary material). Using STING_CRISPR and the SHAP TreeExplainer model [56], we obtain SHAP values ϕ for all PDB snapshots in the ML dataset, where $\phi _j^{(i)}$ denotes the SHAP value assigned to the *j*th feature for the *i*th datapoint. We also obtain the SHAP importance of each features in STING_CRISPR, where the SHAP importance of the *j*th feature is given by $I_j = \frac{1}{|D|} \sum _{i=1}^{|D|} \phi _j^{(i)}$.

Each input feature in STING_CRISPR has the following six properties: an associated Cas9 residue, Cas9 domain, contiguous Cas9 domain, parent descriptor class, (neighbour) descriptor class, and neighbour aggregation method. For example, the feature Cas9_733_neighbours_side_chain_angle_3_VD has properties Cas9 residue 733, Cas9 domain RuvC, contiguous Cas9 domain RuvC-II, parent descriptor class SCO, descriptor class side chain orientation with VD (SCO-VD), and neighbour aggregation method VD. Since we can group features in STING_CRISPR by a certain property, count the number of features in each feature group, and compute the SHAP importance $I_J = \frac{1}{|D|}\sum _{i=1}^{|D|} |\sum _{j \in J} \phi _j^{(i)}|$ of each feature group *J*, we compute feature counts and SHAP importances for each of the feature groups arising from each of the aforementioned six properties, and subsequently use bar plots for data visualization.

Cas9 residues appearing far apart in the sequence space may actually be spatially proximal in the Cas9 complex. In light of this, to identify the residue clusters (i.e. hotspots) found by our training procedure, we measure the pairwise distances between two residues in STING_CRISPR averaged across the 672 PDB snapshots, and subsequently use Seaborn’s clustermap algorithm to create the clusters, while setting a maximum distance of 12 Å for any two residues within the same cluster. Based on these residue clusters, we compute the feature counts and SHAP importance of each residue cluster, with residues in STING_CRISPR not belonging to any residue cluster placed into the ‘other’ residue group. To gain spatial intuition, we use PyMOL [57] to visualize the residue clusters. Specifically, we use the last PDB snapshot from the on-target trajectory CMUT1 for visualization. For each residue-base combination formed between the STING_CRISPR residues and heteroduplex bases, we also count the number of PDB snapshots where the residue’s α-carbon atom is 3 to 7 Å away from the heteroduplex base’s C4’ atom, and use heatmaps to visualize the counts.

### Evaluation of the structural impact of the mutations

The impact of the tsDNA mutations on the overall dynamics of the system structure was evaluated by performing a parametric analysis of the stability of the most relevant residues/bases of the system. The considered parameters are average and standard deviation of the RMSD with respect to the initial conformation. Under normality assumption, the Kullback–Leibler divergences between the RMSD distributions of the residues which emerged as the most informative from the ML analysis as well as those of the bases involved in the heteroduplex complex were calculated considering as a reference the trajectory of the CMUT1 system, data shown in the Supplementary material. This allows to immediately pinpoint the sites where the difference in behavior is maximal. After doing this, a more detailed distinction was performed, separating the sites differing because of being more mobile from those differing because of being more stable.

## Results

### Structural determinants of cleavage activity

#### Consistency with the latest experimental structures

As more thoroughly described in the ‘Materials and methods’ section, our starting structure, referred to as CMUT1 (see Fig. 1C), was derived from the closest entry of the sequence database to the available structures including also the DNA and the SpCas9 (referred to as Cas9 onwards) counterparts. This structure is complete and conformationally consistent with the catalytically active structure published in [35], PDB code 6O0Y. In order to expand our analysis, we included in our evaluations also the structure published in the work by Bravo *et al.* [7] (PDB code 7S4X). In the latter work, catalytically active conformations of Cas9 in presence of mismatches were determined through kinetics-guided cryo-EM. Therefore, we also decided to check that the key structural features reported in this work are reflected in our analysis. Four structural features of the 7S4X structure are shown by the authors to be significant for its catalytic activity:

- Kinkedness of the RNA/DNA heteroduplex (residues B1–15 D1–20 in 7S4X; C14–30 B2–17 in CMUT1)—this characteristic is shared;
- Conformation of the L1 loop (residues A765–780 in 7S4X and in CMUT1)—the conformations are virtually identical;
- Conformation of the L2 loop (residues A906–918 in 7S4X and in CMUT1)—average heavy atom RMSD against 250 ns CMUT1 MD trajectory: 3.8 Å; and
- Conformation of the RuvC loop (residues A1010–1030 in 7S4X and in CMUT1)—average heavy atom RMSD against 250 ns CMUT1 MD trajectory: 3.8 Å.

#### Mismatch-induced dynamical effects

We challenge the idea that a single PAM-distal mismatch between the sgRNA and the tsDNA always destabilizes the system. This is done by comparing the RMSD distributions along the dynamics of individual sites, i.e. protein residues or sgRNA/tsDNA bases, with respect to the corresponding distributions obtained from the dynamics of the reference structure CMUT1, which has no mismatch. Summarizing the results, which are detailed in the Supplementary material, we can say that point mutations in the tsDNA result in a local destabilization of the sgRNA bases in the PAM-distal region, where they are located, but seem also to stabilize some RNA bases in the PAM-proximal region and induce a remarkable stabilization, quantified by the RMSD standard deviation along the trajectories, of some tsDNA bases, again in the PAM-proximal region. This finding could explain why some PAM-distal point mutations lead to increased cleavage activity. Furthermore, some degree of stabilization is observed in some Cas9 residues emerging as important from our ML approach, as shown in the stability analysis results included in the Supplementary material. The finding also corroborates with the positive correlation (Spearman: 0.418, Pearson: 0.503) found between heteroduplex base pair distance sums, a quantity informative on the overall stability of the guide RNA–tsDNA heteroduplex, and CRISPR–Cas9 cleavage activities (see Supplementary Fig. S8). In summary, this analysis shows that the local destabilization induced by a single mismatch between the sgRNA and the tsDNA in the PAM-distal region can be compensated by the stabilization in other nearby positions. A possible explanation of such compensation is further elaborated in the ‘Discussion’ section.

### Test performance and model interpretation of STING_CRISPR

**Figure 3.**
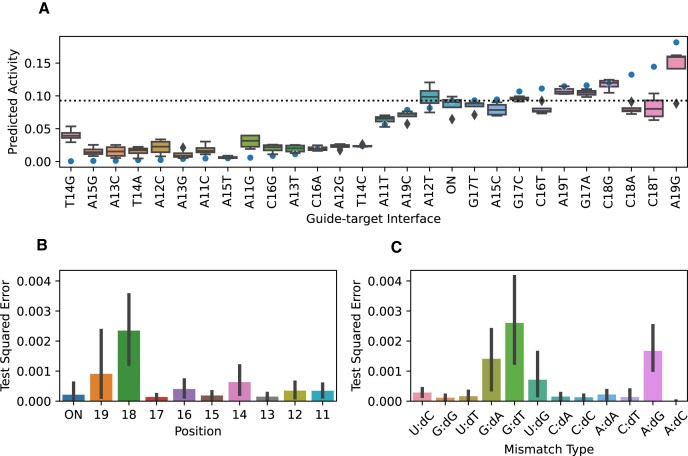
Test performance of STING_CRISPR. (**A**) STING_CRISPR’s predicted cleavage activities for the hold-out test set containing the last 4 snapshots from each of the 28 MD trajectories. Blue dots indicate experimental cleavage activity labels for the 28 interfaces. Guide–target interfaces listed on the *x*-axis are sorted by increasing experimental activity. ON = on-target interface. (**B**) STING_CRISPR’s squared error between predicted and actual CRISPR–Cas9 cleavage activity values for snapshots in the test set, categorized by being an on-target interface or a PAM-distal mismatch position. (**C**) STING_CRISPR’s test squared error between predicted and actual CRISPR–Cas9 cleavage activity values for the different off-target mismatch interface types.

On the hold-out test dataset of size 112, STING_CRISPR attains a Spearman correlation of 0.819, a Pearson correlation of 0.916, a mean squared error of 5.92 × 10^−4^, and a mean absolute error of 1.68 × 10^−2^, demonstrating high model performance and affirming that residue-resolved physico-chemical/structural features can be utilized for CRISPR–Cas9 cleavage activity prediction. Ordered by increasing cleavage activity, we can see that there is minimal difference between the predicted and actual cleavage activities across all guide–target interfaces in this study (Fig. 3A) apart from base mutations T14G, C18A, C18T, and A19G with extreme levels of cleavage activity. Such an observation is corroborated by high test square errors in positions 14, 18, and 19 (Fig. 3B) and mismatch interface types G:dA, G:dT, U:dG, and A:dG (Fig. 3C).

Using various physico-chemical and structural descriptors, the 30 residue-resolved input features of STING_CRISPR characterize 23 Cas9 residues. The SHAP summary plot generated from STING_CRISPR using all 672 conformations shows Cas9_733_neighbours_side_chain_angle_3_VD as the most important feature in STING_CRISPR, where increasing its feature value increases predicted cleavage activity (see Supplementary Fig. S2). Through hierarchical clustering of pairwise residue distance calculations between the C-α atoms of these 23 residues (see Fig. 5A), we see that 17 of the 23 residues form following 4 residue clusters:

- Group 1 with residues 1016 and 1017;
- Group 2 with residues 728, 730, 732, 733, and 734;
- Group 3 with residues 837, 838, and 839; and
- Group 4 with residues 136, 164, 317, 402, 408, 411, and 415,

which are coloured red, orange, pink, and yellow, respectively (see right part of Fig. 2B and Fig. 5F and G). Such localization of residue clusters likely indicates some biological, functional, constitutive, or structural importance within those regions. For completeness, we also group the remaining six residues 268, 908, 919, 1010, 1025, and 1122 to form the ‘other’ residue group (coloured light blue).

**Figure 4.**
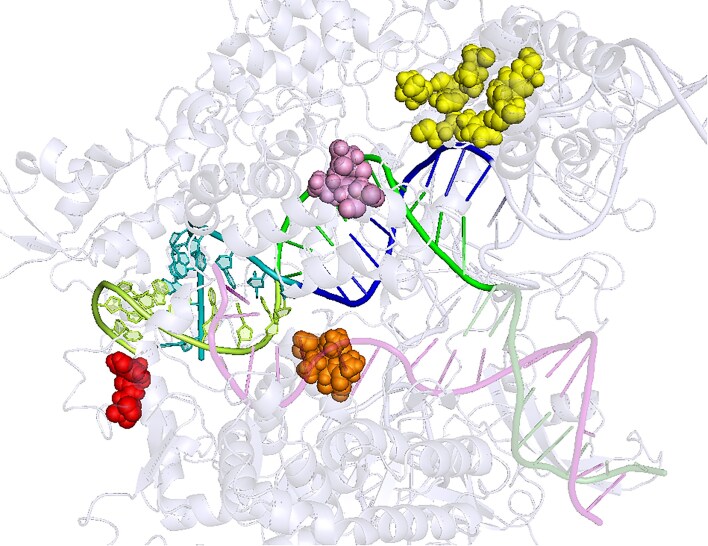
PyMOL cartoon visualization of the sgRNA–dsDNA–Cas9 complex, taken from the last (i.e. 24th) snapshot of CMUT1’s MD trajectory. Shown as spheres, the four CRISPR–Cas9 residue clusters 136/164/317/402/408/411/415, 728/730/732–734, 837–839, and 1016/1017 are highlighted in yellow (top right), orange (center bottom), pink (center top), and red (bottom left), respectively. Other parts of the Cas9 are visualized as grey ribbons. Shown as ribbons, the colour scheme is as follows for non-Cas9 components: PAM-distal sgRNA = teal, PAM-proximal sgRNA = blue, PAM-distal target DNA strand = limon, PAM-proximal target DNA strand = green, and non-target DNA strand = transparent purple.

**Figure 5.**
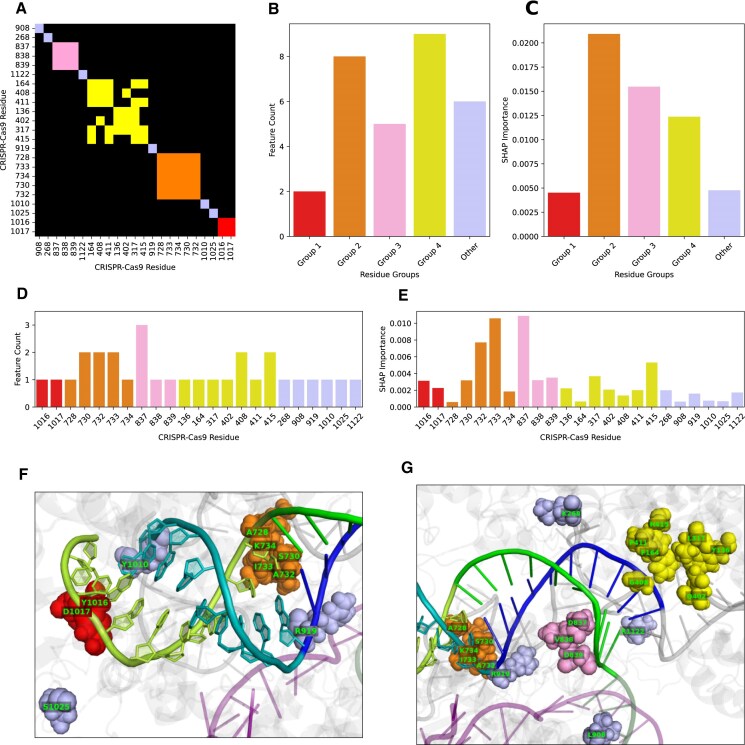
The ML pipeline identifies four residue clusters, namely Group 1 (residues 1016/1017, coloured red), Group 2 (residues 728/730/732–734, coloured orange), Group 3 (residues 837–839, coloured pink), and Group 4 (residues 136/164/317/402/408/411/415, coloured yellow). The fifth group ‘other’ consists of residues identified by the pipeline that do not belong to the above clusters (residues 268/908/919/1010/1015/1122, coloured light blue). (**A**) Binarized hierarchically clustered heatmap for the 23 Cas9 residues identified by the ML pipeline. Heatmap cells for residue pairs whose *C*_α_ atoms are <12 Å apart are coloured according to their associated residue groups, and black otherwise. Feature counts (**B**) and SHAP importances (**C**) of the five residue groups. Feature counts (**D**) and SHAP importances (**E**) of the 23 important Cas9 residues, with residues grouped and coloured by the five residue groups. PyMOL cartoon visualization of the PAM-distal (**F**) and PAM-proximal (**G**) portions of the sgRNA–dsDNA heteroduplex taken from the last (i.e. 24th) snapshot of CMUT1’s MD trajectory. Shown as labelled spheres, the 23 CRISPR–Cas9 important residues are coloured by their residue groups. Shown as ribbons, the colour scheme of other components is as follows: other parts of Cas9 = grey, PAM-distal sgRNA = teal, PAM-proximal sgRNA = blue, PAM-distal target DNA strand = limon, PAM-proximal target DNA strand = green, and non-target DNA strand = transparent purple.

Using these five residue groups, we see high feature counts and SHAP importances for Groups 2 and 4 (Fig. 5B and C), showing that Groups 2 and 4 significantly contribute to STING_CRISPR’s predicted cleavage activity. As for the feature counts of 23 residues, we see that most residues only have one feature, with residue 837 having the highest feature count of 3 (Fig. 5D). SHAP importances vary widely between the 23 residues, with residues 733 and 837 having the highest SHAP importances. Specifically, residues 1016, 733, 837, and 415 have the highest SHAP importances in residue Groups 1–4, respectively.

The residue clusters are spatially located next to different parts of the heteroduplex, and come from various Cas9 domains (Figs 4 and 5F and Supplementary Fig. S7). Specifically, Group 1 consists of RuvC residues located in the PAM-distal part of the heteroduplex, Group 2 consists of RuvC residues located at the midde part of the heteroduplex, Group 3 consists of HNH residues located at the catalytic site which cuts the tsDNA, and Group 4 consists of Rec I residues located on the sgRNA side of the PAM-proximal portion of the heteroduplex. As for the other residues, residues 1010 and 1025 flank Group 1 on the sgRNA and tsDNA sides, respectively. Located in the middle part of the heteroduplex, residue 919 is also spatially close to residue Group 2. Using a similar approach, we also see that the four residue clusters draw features from different parent descriptor classes, which have varying SHAP importances in the different residue clusters (see Supplementary Fig. S6).

**Figure 6.**
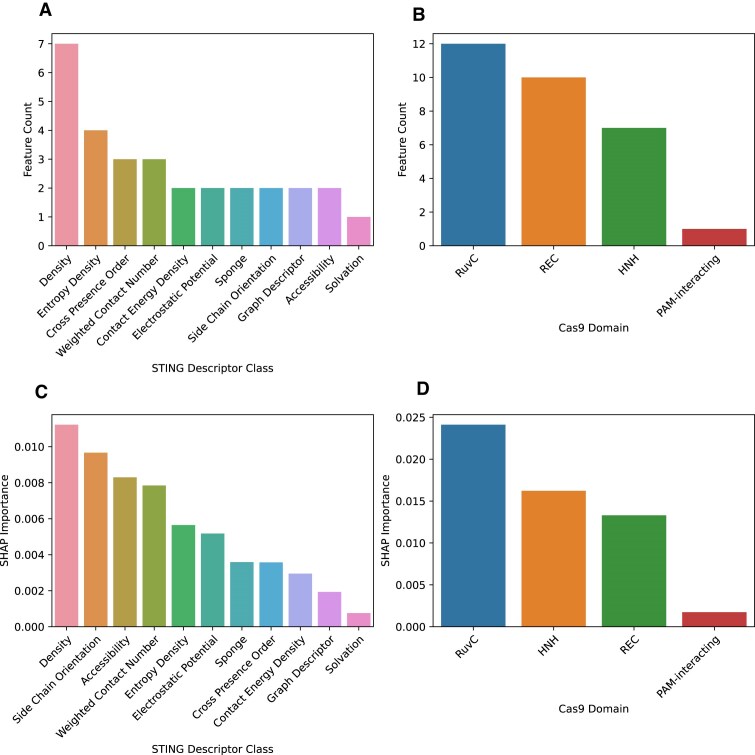
(Top) STING_CRISPR’s feature counts categorized by STING descriptor classes (**A**) and CRISPR–Cas9 domains (**B**), respectively, sorted by decreasing count. (Bottom) STING_CRISPR’s SHAP importance values for STING descriptor classes (**C**) and CRISPR–Cas9 domains (**D**), respectively, sorted by decreasing SHAP importance. Only STING descriptor classes or Cas9 domains with non-zero count or SHAP importance are shown.

To varying degrees, predictions made by STING_CRISPR are influenced by the different parent descriptor classes and Cas9 domains associated with the 30 input features. In terms of parent descriptor classes, density, entropy density, and cross presence order have the most features, and density, SCO, and accessibility have the highest SHAP importances (Fig. 6). In terms of Cas9 domains, RuvC is shown to have the highest feature count and SHAP importance among the RuvC, HNH, REC, and PIs. In a similar fashion, feature count and SHAP importance analysis of the four neighbour aggregation methods show that SW and VD have high feature counts and SHAP importances (Supplementary Fig. S3). The same analysis but for descriptor classes show that density with SW has highest count, but SCO with VD and accessibility have the highest SHAP importance.

When considering all 672 atomistic model conformations, all residues apart from 411 and 733 are surface residues, but only residues 136, 164, 268, 402, 408, 728, 730, 919, 1016, and 1122 are interface residues according to Surfv, NACCESS, and NSC (Supplementary Fig. S4). In addition, in the 672 conformations, most residues are surface residues (Supplementary Fig. S5A), and on average there are around 12 interfaces residues in a given conformation (Supplementary Fig. S5B). In terms of SHAP importances, we see that surface residues have a much higher SHAP importances than non-surface residues (Supplementary Fig. S5D), and that interface residues have less SHAP importance than non-interface residues (Supplementary Fig. S5E). Averaged across the 672 PDB snapshots, 55.6%, 60.5%, and 52% of the residues among the four residue clusters are residues located at the interface between Cas9 and the R-loop complex (i.e. interface residues), according to SurfV, NACCESS, and NSC, respectively. When rerunning the training procedure to train on residues 3–1363 instead of just the HPRs, we find that both the feature count and the SHAP importance of HPRs are higher than those of non-HPRs (Supplementary Fig. S5C and F).

### Test performance when generalizing to unseen guide–target interfaces

**Figure 7.**
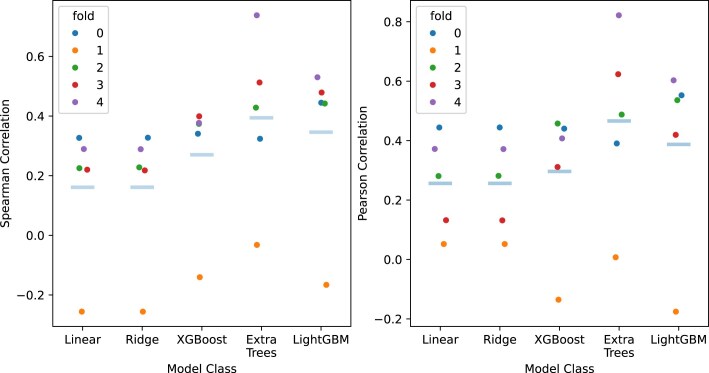
Five-fold cross-validation Spearman (left) and Pearson (right) correlation performance when using linear regression, ridge regression, XGBoost, extra trees, and LightGBM. Test sets for each cross-validation fold was constructed by binning snapshots associated with the trajectory with the *n*th lowest cleavage activity into the test partition of fold $n\mod {5}$, and into the training partition in the other folds. Light blue horizontal line represents the mean correlation across the five folds.

**Figure 8.**
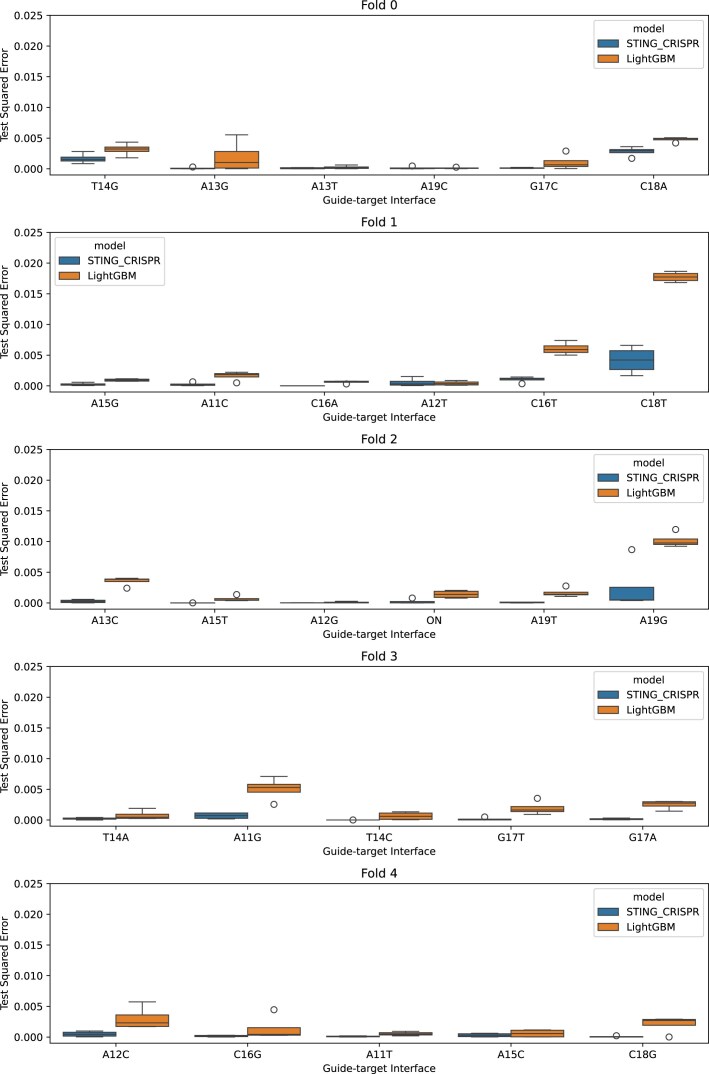
Box plots comparing test squared errors between STING_CRISPR and the new LightGBM model trained in Fig. 7. The *x*-axis lists the guide–target interfaces held-out in each of the five cross-validation folds. Circles represent outliers in the box plot.

We also tried withholding snapshots from entire sgRNA–target pair trajectories instead of the last four snapshots, as holding out sgRNA–target pairs would serve as a better test for evaluating the ML model’s ability to generalize to unseen sgRNA–target pairs—an ability observed in many existing ML-based off-target activity prediction tools. However, the test performance varies across the five folds in five-fold cross-validation when a variety of ML models without feature selection (linear regression, ridge regression, XGBoost, extra trees, and LightGBM) are used (see Fig. 7). As seen in the figure, all ML model types fail to generalize on fold 1. Examining the distribution of test squared errors per sgRNA–target pair in the LightGBM model, we observe variance in predicted activities within a sgRNA–target pair MD trajectory, indicating variability between snapshots within the trajectory (see Fig. 8). Owing to poor test performances, we do not proceed with SHAP interpretation of these ML models. Details on methods can be found in the Supplementary material under the section ‘Holding out trajectories as test sets’.

## Discussion

### Structural plasticity of the heteroduplex: structural stability of mismatches

According to our MD simulations, introducing a mismatching mutation in the PAM-distal region of the tsDNA does not necessarily produce a major structural instability in the overall structure of the heteroduplex nor in that of the Cas9 protein. By using the analysis described in the Supplementary material, we actually found that these mutations produce minor perturbations in the dynamics of the sgRNA in the PAM-distal region, but also, unexpectedly, a stabilizing effect on some RNA bases in the PAM-proximal region and on some residues of the Cas9 protein. This is consistent both with the experimental cleavage activity data and with the observations concerning the heteroduplex base pair distance sums. We suspect such stabilizing effect arises from a release of mechanical strain in the heteroduplex, where the mechanical strain originates from differing helical parameters between RNA–DNA heteroduplexes (closer to A-form than B-form) and A-form RNA or B-form DNA duplexes [58, 59].

### Nanoenvironment approach

At this point, it is imperative to emphasize that the concept ‘nanoenvironment’ is referred hereto as a specific internal protein region, with well-defined characteristics and a unique set of corresponding STING descriptors [43–46] that are able to select only the amino acid residues that make up that part of the protein region. Previously, we named such functionally distinct regions as protein districts, using a common analogy of internal protein regions with city districts. Previous work [43–47, 60] has been successfully connected to the similar characterization of certain residues within a protein region with some functional properties (such as enzyme activity or protein interfaces) of the system in the study. In this work, we identified four specific hotspots (residues 136/164/317/402/408/411/415; 730/732–734; 837–839; and 1016–1017) which are borderline with the interface between the Cas9 protein and the heteroduplex. Namely, approximately half of the hotspot residues are part of the protein–heteroduplex interface [formed by the interface forming residues (IFRs)] and the other half belong to the immediate next layer leaning on the IFRs. Those hotspots are actually groups of amino acid residues to which specific STING descriptors [43–47, 60] are attached. The localization of amino acid residues within hotspots is indicative of their functional importance in terms of modulating off-target cleavage activity. To get the location of hotspots, however, it was first necessary to obtain a list of features by the already described computational protocol.

### Cleavage activity prediction models and their interpretability

Some of the most successful models for CRISPR–Cas9 off-target activity prediction are based on DL and managed to reach high predictive performance in terms of classification [9, 10, 18, 28]. The building of sufficiently accurate regression models for the problem of off-target cleavage activity prediction is still an open challenge in spite of the increasing sophistication of DL approaches and encoding practices applied on the sgRNA–tsDNA (guide–target) sequence pair [9, 10, 18]. A recent advance utilized structural information of the guide–target sequence pair extracted from MD simulations in order to construct RNA–DNA molecular interaction fingerprints, i.e. structurally informed encodings of the guide–target heteroduplex [61]. However, none of the previous works leveraged the information from the entire CRISPR–Cas9 complex, especially from the Cas9 protein. The current state of the field suggests that it has reached its possible best performance on this type of learning problem associated with mainly describing a datapoint with a guide–target sequence pair or a structurally inspired heteroduplex encoding from it.

As an alternative to proposing another new learning model on existing datasets based on guide–target sequence pairs, our work proposes a new learning approach/problem that takes into account the whole sgRNA–dsDNA–Cas9 complex in its entire physico-chemical/structural internal ‘reality’. This is achieved by obtaining a set of physico-chemical/structural features characterizing all guide–target proximal residues in a given sgRNA–dsDNA–Cas9 complex that accommodates a given guide–target pair. Unlike Chen *et al.* [61], our physico-chemically/structurally informed features are obtained from MD simulation of the entire CRISPR–Cas9 complex, which includes the Cas9 protein in addition to the guide–target heteroduplex and other parts of the R-loop.

We work under the assumption that the 3D internal protein nanoenvironments, and features therein, of guide–target pairs are able to provide an information-rich representation of the guide–target pairs themselves. We therefore trained an ML pipeline with a built-in feature selection step, i.e. scikit-learn’s SelectFromModel, in order to simultaneously identify the most important features informative for cleavage activity prediction and train an ML model which predicts cleavage activities. We then evaluate the ML model’s ability to predict cleavage activities for unseen 3D protein nanoenvironments (associated with guide–target pairs) in the test set. Our results indicate that the trained model successfully captures the relationship between 3D protein nanoenvironments and cleavage activities for the studied sgRNA–tsDNA pairs. In particular, the trained model is capable of predicting experimental cleavage activities with an accuracy of 0.819 Spearman and 0.916 Pearson correlation coefficients. While this delivers a high level of accuracy, the current model presented in this study was only trained on a small subset of experimentally available sgRNA–tsDNA pairs. Another limitation of our approach is that the activity prediction of any unseen sgRNA–tsDNA pair would require performing a new MD trajectory. Therefore, the current model is not expected to replace existing high-throughput methods aiming at predicting off-target cleavage activity at the genomic scale for any sgRNA–tsDNA pair.

However, the advantage of our method consists in leveraging often neglected factors such as features related to Cas9 residues influencing off-target activity. These features are descriptors characterizing a particular residue. We found that the parent descriptor classes in order of decreasing SHAP importance are: density, SCO, accessibility, weighted contact number, entropy density, electrostatic potential, sponge, cross presence order, contact energy density, graph descriptor, and solvation. Our analysis also identifies the most significant residue hotspots 136/164/317/402/408/411/415, 730/732–734, 837–839, and 1016–1017 responsible for modulating cleavage activity for the studied sgRNA–tsDNA pairs. Our study highlights the importance of more general characteristics than mere residue identity. The most important residues identified in this work are in fact carriers of important characteristics rather than pure amino acid properties. Furthermore, we found that general determinants of internal protein packing is of fundamental importance and this is obvious from the presence of descriptors such as density, sponge, and weighted contact number. In addition, general geometry (accessibility), physico-chemical features (electrostatic potential), and finally the evolutionary preservation of sequences (entropy density) are pertinent and crucial for the determination of cleavage activity for the studied sgRNA–tsDNA. Further studies are needed in order to establish whether our findings still apply for any sgRNA–tsDNA pair such as ones containing multiple PAM-distal or PAM-proximal mismatches and for any sgRNA. While these investigations are not in the scope of our current proof-of-concept study, the agreement with experimental findings are encouraging.

The identity of residues in some of the residue hotspots is in concordance with recent experimental findings. For example, residue 837 has been hypothesized to aid in the positioning of the target DNA relative to the HNH domain [62] and to function as a catalytic residue [63, 64], although the latter hypothesis has been questioned by more recent findings [62]. Along with 837, residues 838 and 839 are of known importance as parts of the catalytically active site of the HNH domain, coordinating the metal ions [62, 65]. Indeed, the mutation D839A was shown to compromise gene editing activity in site-directed mutagenesis experiments [62]. Proximal to 402 and 408, residue 406 is part of the negative pocket of the REC-I domain which is instrumental in RNA recruitment [66]. Residues 1016 and 1017, together with residues 1010 and 1025 detected by STING_CRISPR, are part of a RuvC loop which was shown to only stabilize PAM-distal mismatches in the heteroduplex rather than activate on-target interfaces [7]. In addition to these residues, our analysis characterizes Cas9 residues 268, 908, 919, and 1122 as important residues. Interestingly, residues 908 and 919 are part of the L2 loop, which interacts with the ntsDNA in order to dock HNH to the tsDNA, i.e. activate the HNH domain [67], and reposition the ntsDNA in the RuvC cleavage site [7]. Residue 908 also interacts with the unwound DNA in cases of multiple PAM-distal mismatches, thereby hampering HNH cleavage activation [68], though 908 is not shown to interact with the PAM-distal region in the 672 PDB snapshots. Residue 268 detected by STING_CRISPR is next to residues 267 and 269, both of which were shown to form contact with target strand that kink the ntsDNA [69].

The approach we took in this paper would be also capable of predicting the effect of certain residue mutations on cleavage activity for sgRNA–tsDNA pairs including, but not limited to, the ones covered by this work. Such an approach would be similar to Venanzi *et al.* [70]’s approach in using MD simulation-derived features for enyzme variant activity prediction. In fact, the present model is already fully functional in this regard since it has learned the relationship between the protein 3D nanoenvironments of guide–target pairs and cleavage activities and is, therefore, capable of making a prediction of cleavage activity based on the protein 3D nanoenvironment of a guide–target pair irrespective of ‘how the protein 3D nanoenvironment is realized’. Therefore our trained model already has the ability (by construction) to predict the effect of any Cas9 residue mutation on (off-)target cleavage activity provided that the protein 3D nanoenvironment of corresponding guide–target pair is computed consistently via MD. This later task can be automated following the same steps outlined in Fig. 1 but using the initial systems in which Cas9 has the desired residue mutations. While the aim of the paper was not to predict the effect of residue mutations on (off-) target cleavage activity, our proposed approach also offers a possible computational solution to tackle this important and very timely problem. This type of computational approach would pave the way for *in silico* design of optimal 3D protein nanoenvironments of desired guide–target pairs (representing optimal combination of mutations of Cas9) that would maximize on-target activity and minimize off-target effects.

The current limitations of our approach include the necessity of performing a MD trajectory in order to generate the protein 3D nanoenvironment for a given sgRNA–tsDNA pair. Therefore, our approach is not expected to compete with the currently available state-of-the-art methods [9, 13, 17, 61, 71, 72] for predicting off-target activity for any sgRNA–tsDNA pair.

### Limitations

The current limitations of our approach include the necessity of performing a MD trajectory in order to generate the protein 3D nanoenvironment for a given sgRNA–tsDNA pair. Therefore, our approach is not expected to compete with the currently available state-of-the-art methods [9, 13, 17, 61, 71, 72] for predicting off-target activity for any sgRNA–tsDNA pair.

The 23 Cas9 residues found in this study are important only for the 28 ‘studied sgRNA–tsDNA pairs’, rather than for all possible SpCas9 guide–target interfaces. While the 28 sgRNA–tsDNA pairs are all annotated with experimental (off-)target cleavage activities measured in Jone Jr *et al.* [73], we acknowledge that data from further experimental biochemical assays could help to (in)validate the 23 Cas9 residues identified in STING_CRISPR, thus allowing one to assess the extent to which STING_CRISPR is able to identify Cas9 residues which significantly modulate cleavage activity (e.g. via precision/recall scores). For example, one could perform alanine scanning at the 23 Cas9 residues for all 28 studied sgRNA–tsDNA pairs and measure experimental cleavage activities for the 23*28 combinations. However, such an experiment is beyond the scope of this study.

Nonetheless, in the previous subsection, we have been able to relate 8 of the 23 Cas9 residues to the existing literature, which highlight the importance of these 8 residues. Furthermore, the assessment of Cas9 residue importance in cleavage activity via ML model interpretation is unprecedented. Based on the above two statements, we believe that this provides sufficient evidence for STING_CRISPR to lay the foundations for a new type of interpretable ML models which account for the ways in which Cas9 residues affect cleavage activity.

We also tried withholding snapshots from entire sgRNA–target pair trajectories instead of the last four snapshots, as holding out sgRNA–target pairs would serve as a better test for evaluating the ML model’s ability to generalize to unseen sgRNA–target pairs. However, the test performance varies across the five folds in five-fold cross-validation when a variety of ML models without feature selection (linear regression, ridge regression, XGBoost, extra trees, and LightGBM) are used (see Fig. 7). Examining the distribution of test squared errors per sgRNA–target pair in the LightGBM model, we observe variance in predicted activities within a sgRNA–target pair MD trajectory, indicating variability between snapshots within the trajectory (see Fig. 8).

Regarding model performance in Fig. 7, we acknowledge that all ML models fail to generalize in fold 1. This is likely because the data used for ML model training does not contain sgRNA–target mismatch interfaces which cover all base pair positions and mismatch types. This issue could easily be resolved by including trajectories of guide–target interfaces with multiple mismatches in the ML dataset. In particular, one would ensure that all heteroduplex base pair positions are covered in the training set while making sure that there are no overlapping guide–target interfaces between the training and test sets (to avoid data leakage). Nonetheless, such a proposal is beyond the scope of this study due to computational resources.

## Conclusions

Research efforts and applications using CRISPR–Cas9-based genome engineering have been increasing since the discovery of the CRISPR–Cas9-based ‘genetic scissors’, which has transformed industrial biotechnology and modern agriculture. CRISPR–Cas9-based genome engineering shows great promise for curing diseases with an unparalleled efficiency that would have been inconceivable at the beginning of the century. However, its ability to transform medicine strongly relies on the understanding of possible side effects caused by the off-target activity of the CRISPR–Cas9 gene editing system. This research challenge catalyzed tremendous efforts in both experimental and computational sciences. As a result, the most successful computational models, which are based on deep neural networks or biological fingerprinting, managed to deliver accurate results in the activity classification of guide–target sequence pairs but interpreting these models does not deliver information on the importance of Cas9 residues in modulating cleavage activity. Therefore, building accurate and explainable models that facilitate the design of CRISPR–Cas9-based gene editing experiments is among the greatest challenges of present-day computational biology.

This work is one step forward towards meeting this challenge and introduces a reformulation of the learning task for CRISPR–Cas9 off-target cleavage activity prediction with the ultimate goal of building explainable ML models capable of predicting CRISPR–Cas9 off-target cleavage activity with high accuracy. The contributions of this work are as follows:

- Successfully deriving a novel and powerful ‘physico-chemical and structural’ information-enriched representation for guide–target sequence pairs consisting of 30 features (capturing the protein 3D nanoenvironment of the guide–target pair);
- Training an ML model to learn the relationship between the said representation and the off-target cleavage activity; and
- Shedding light on the structural and physico-chemical determinants of CRISPR–Cas9 off-target cleavage activity and identifying the most important residues, whose structural and physico-chemical descriptors modulate (off-)target activity for the studied sgRNA–tsDNA pairs, by interpreting the successful ML predictions.

For the first time, our ML model STING_CRISPR is also capable of predicting the effect of CRISPR–Cas9 residue mutations on off-target cleavage activity, paving the way for further exploration and discoveries.

## Supplementary Material

lqaf054_Supplemental_Files

## Data Availability

Structural stability analysis summary is reported in the Zenodo repository (DOI: 10.5281/zenodo.11473926). TSV files containing STING descriptor values are reported in the Zenodo repository (DOI: 10.5281/zenodo.11472743). Sample Python scripts for using Nanoenv-Cas9-WNA are available at https://github.com/jeffmak/crispr-cas9-nanoenv (Zenodo; DOI: 10.5281/zenodo.14210188). PDB snapshots arising from the 28 trajectories in this study are available as supplementary information.
