# Supplementary material for: Learning to utilize internal protein 3D nanoenvironment descriptors in predicting CRISPR–Cas9 off-target activity
Source: NAR Genom Bioinform. 2025 May 21;7(2):lqaf054. doi: 10.1093/nargab/lqaf054 (PMC12093099; doi:10.1093/nargab/lqaf054)
Supplement: lqaf054_Supplemental_Files [file lqaf054_supplemental_files.zip › suppl.pdf]

Supplementary material for “Learning to utilize internal protein 3D nanoenvironment descriptors in predicting CRISPR-Cas9 off-target activity”

Jeffrey Mak,[1†] Artemi Bendandi,[2] José Augusto Salim,[3]
Ivan Mazoni,[4] Fabio Moraes,[5] Luiz Borro,[6]
Florian Störtz,[1] Walter Rocchia,[2‡†] Goran Neshich,[4‡†] Peter Minary[1‡†]

[1]Department of Computer Science, University of Oxford, Parks Road, Oxford OX1 3QD, UK
[2]CONCEPT Lab, Istituto Italiano di Tecnologia, Via Melen – 83, B Block, 16152 - Genova, Italy
[3]Department Of Plant Biology, Institute of Biology, University of Campinas – UNICAMP, SP, Brazil
[4]Computational Biology Research Group, Embrapa Digital Agriculture, Campinas, SP, Brazil
[5]Physics Department, Institute of Biosciences, Languages, and Exact Sciences (IBILCE), São Paulo State University (Unesp), São José do Rio Preto, SP, Brazil
[6]Claro beOn, São Paulo, SP, Brazil

[†]To whom correspondence should be addressed; E-mail: jeffrey.kelvin.mak@cs.ox.ac.uk, gneshich@gmail.com, walter.rocchia@iit.it, peter.minary@cs.ox.ac.uk.
[‡]These authors are equally credited for conceiving and managing the work from their respective institutions.

STING descriptors

Here we provide a brief description of STING descriptors.

Accessibility

The Amino acid accessibility is calculated according to SurfV (*1*) program. We calculated five different values for accessibility: i) for the protein chain in isolation, ii) for the protein chain in complex with whatever other chain (if) present in the same PDB file and finally, iii) a relative accessibility, iv) a difference between accessibility in isolation and in complex (interface residues), v) a buried surface area (bsa). Details described at `http://sms.cbi.cnptia.embrapa.br/SMS/STINGm/help/solvent_accessible_area.html`. Other flavors of accessibility, such as calculated by Surface Racer (*2*) (a program already used by Blue Star STING for the calculation of Curvature) and NACCESS© (*3*) are also available at the STING RDB2. Additionally, the Shrake-Rupley accessibility (NSC) is also available for analysis (*4*). These algorithms calculate an approximate accessible surface and therefore the corresponding accessibility is also an estimation; this is mainly due to the fact that obtaining precise surface description by those methods is computationally prohibitive, so different algorithms can result in different values of accessible area. Consequently, having all those accessible area values obtained using several algorithms allows one to compare and even define a consensus between the accessibility values of an atom or a residue.

List of accessibility descriptors:

1. Accessibility in isolation using SurfV, NACCESS and NSC: `acc_isol_surfv`, `acc_isol_naccess` and `acc_isol_nsc`,

2. Accessibility in complex using SurfV, NACCESS and NSC: `acc_complex_surfv`, `acc_complex_naccess` and `acc_complex_nsc`,

3. Relative accessibility (RSA) calculated as the ratio between the accessibility in isolation and the absolute solvent accessible area for specific residue type (acc^t_{max}) given in `http://sms.cbi.cnptia.embrapa.br/SMS/STINGm/help/solvent_accessible_area.html`: `acc_rsa_surfv`, `acc_rsa_naccess` and `acc_rsa_nsc`

$$acc\_rsa = \frac{acc\_isol}{acc^t_{max}} \quad (1)$$

4. Difference between accessibility in isolation and in complex (IFR): `acc_ifr_surfv`, `acc_ifr_naccess` and `acc_ifr_nsc`. Residues which have IFR lesser than accessibility in isolation are present in the protein's interface.

5. Buried Surface Area (BSA) is a measure of the size of the interface in a protein-protein complex. BSA is calculated as the ratio between the interface accessible area of a residue and its accessible area in isolation: `acc_bsa_surfv`, `acc_bsa_naccess`, `acc_bsa_nsc`

$$acc\_bsa = \frac{acc\_ifr}{acc\_isol} \quad (2)$$

The absolute (or maximum) accessibility is calculated for each amino acid type using four arbitrarily selected protein structures from PDB, where the selected amino acid type (one of 20) must be located at the C-terminal end. Then, we edited these structures so that the C-terminal residue would be left isolated in the "vacuum" (all other amino acid residues were deleted from the file). The SurfV software is then used to calculate the accessible surface area of the edited structure and among those 4 structures (for each of the 20 amino acids) we identified the maximum and minimum value for the ASA. The difference (rounded to the higher integer number) between the two values (taken percent-wise) was added to the higher of two ASA values. We believe that this is much more convenient than taking the tabular data available in the literature, as the former one is a more realistic approach, based on the real experimental data within the environment of interest — a protein crystal.

Contact energy density

Amino acid contacts in terms of atomic interactions are essential factors to be considered in the analysis of the structure of a protein and its complexes. Residue-residue contacts are calculated according to the description given by Mancini et al. (*5*). Contact Energy Density (CED) of internal protein contacts is then defined as the sum of contact energies (`https://www.cbi.cnptia.embrapa.br/SMS/STINGm/help/energy_contacts_table.html`) of the contacts established within a given sphere, among residues belonging to the same protein chain, and then divided by the volume of the probe sphere. Additionally, we also calculate the CED descriptors using the protein complex which include interchain contacts (CED IFR). For each residue in a protein we calculate 10 CED descriptors (five using only intrachain contacts and five also including interchain contacts) using five different radii for the probe sphere (3, 4, 5, 6 and 7Å).

$$CED_i = \frac{\sum_{c_t \in C_i^r} E(t)}{V(r)} \tag{3}$$

where C_i is the set of contacts of residue i within the probe sphere of radius r, c is a contact of type t, $E(t)$ is the energy for the contact of type t, and $V(r)$ is the volume of the probe sphere.

List of CED descriptors:

1. CED using only internal contacts with probe spheres of radius 3, 4, 5, 6, 7Å centered at residue's Cα atom: `ced_CA_3`, `ced_CA_4`, `ced_CA_5`, `ced_CA_6` and `ced_CA_7`,

2. CED using only internal contacts with probe spheres of radius 3, 4, 5, 6, 7Å centered at residue's LHA atom: `ced_LHA_3`, `ced_LHA_4`, `ced_LHA_5`, `ced_LHA_6` and `ced_LHA_7`,

3. CED including interchain contacts with probe spheres of radius 3, 4, 5, 6, 7Å centered at residue's Cα atom: `ced_CA_IFR_3`, `ced_CA_IFR_4`, `ced_CA_IFR_5`, `ced_CA_IFR_6` and `ced_CA_7`,

4. CED including interchain contacts with probe spheres of radius 3, 4, 5, 6, 7Å centered at residue's LHA atom: `ced_LHA_IFR_3`, `ced_LHA_IFR_4`, `ced_LHA_IFR_5`, `ced_LHA_IFR_6` and `ced_LHA_7`,

Cross link order

Cross Links are defined as contacts (from five types described above under RESIDUE CONTACTS) established among residues that are far apart in the protein primary sequence, but are close in its 3D fold. The order of cross link is defined as a number of such contacts (cross-links) established among independent stretches of the protein sequence (the size of which varies from 15, 20 to 30 amino acids). Only a single occurrence is counted for the Cross Link Order for a given amino acid residue, even though several such contacts could be observed "aiming" at the same "contacted" sequence stretch. In other words, a central amino acid can make more than one contact with the targeted sequence stretch and each one of those can be established with a different amino acid belonging to that same stretch of probing sequence size (15, 20 or 30 AAs long).

The higher the order, the more important that residue must be for the protein folding/stability/binding. This specific STING descriptor is calculated by varying three input parameters:

- the size of the sequence stretch separating the residues in contact (15, 20 or 30 AA's long),
- the radius size of the probing sphere within which the contacts are counted (3.5, 5 and 8.5 Å), and
- The center of the probing sphere (being either C-α, C-β or Last Heavy Atom in the side chain).

List of CLO descriptors:

1. CLO with probe sphere centered at the C-α for three different stretch lengths (15, 20, and 30 AA's) and three different radii (3.5, 5, and 8.5 Å): `clo_35_15_CA`, `clo_5_15_CA`, `clo_85_15_CA`, `clo_35_20_CA`, `clo_5_20_CA`, `clo_85_20_CA`, `clo_35_30_CA`, `clo_5_30_CA`, `clo_85_30_CA`,

2. CLO with probe sphere centered at the C-β for three different stretch lengths (15, 20, and 30 AA's) and three different radii (3.5, 5, and 8.5 Å): `clo_35_15_CB`, `clo_5_15_CB`, `clo_85_15_CB`, `clo_35_20_CB`, `clo_5_20_CB`, `clo_85_20_CB`, `clo_35_30_CB`, `clo_5_30_CB`, `clo_85_30_CB`,

3. CLO with probe sphere centered at the LHA for three different stretch lengths (15, 20, and 30 AA's) and three different radii (3.5, 5, and 8.5 Å): `clo_35_15_LHA`, `clo_5_15_LHA`, `clo_85_15_LHA`, `clo_35_20_LHA`, `clo_5_20_LHA`, `clo_85_20_LHA`, `clo_35_30_LHA`, `clo_5_30_LHA`, `clo_85_30_LHA`,

Cross presence order

Cross Presence Order (CPO) is defined as a "presence" within a probing sphere (centered at a given residue) of any residue that is far apart in the protein primary sequence from the central residue, but is close in its 3D fold. Remaining details are equivalent to those described above under cross link order.

List of CPO descriptors:

1. CPO with probe sphere centered at the C-α for three different stretch lengths (15, 20, and 30 AA's) and three different radii (3.5, 5, and 8.5 Å): `cpo_35_15_CA`, `cpo_5_15_CA`, `cpo_85_15_CA`, `cpo_35_20_CA`, `cpo_5_20_CA`, `cpo_85_20_CA`, `cpo_35_30_CA`, `cpo_5_30_CA`, `cpo_85_30_CA`,

2. CPO with probe sphere centered at the C-β for three different stretch lengths (15, 20, and 30 AA's) and three different radii (3.5, 5, and 8.5 Å): cpo_35_15_CB, cpo_5_15_CB, cpo_85_15_CB, cpo_35_20_CB, cpo_5_20_CB, cpo_85_20_CB, cpo_35_30_CB, cpo_5_30_CB, cpo_85_30_CB,

3. CPO with probe sphere centered at the LHA for three different stretch lengths (15, 20, and 30 AA's) and three different radii (3.5, 5, and 8.5 Å): cpo_35_15_LHA, cpo_5_15_LHA, cpo_85_15_LHA, cpo_35_20_LHA, cpo_5_20_LHA, cpo_85_20_LHA, cpo_35_30_LHA, cpo_5_30_LHA, cpo_85_30_LHA,

Curvature

The curvature value for each amino acid is calculated using the Surface Racer (*2*) program. Surface Racer calculates the curvature value first for each atom. Then, a curvature of residues is obtained as an average of the surface atoms' curvatures. The curvature is defined at the atomic level, that is, each atom of the protein is assigned a curvature value corresponding to the region where it is located on the molecule's surface. A negative value is assigned to atoms in concave regions, positive for atoms in convex regions and the value zero to the buried atoms (atoms not at the protein surface). To assign values to the amino acid residues of each protein, Blue Star STING calculates the mean curvature considering only the residues on the protein's surface (curvature $\neq 0$). The curvature description is calculated for the chain in isolation and in complex with other chains (if present).

Using Surface Racer program we also calculate the accessible surface area (ASA) and molecular surface area (MSA) for each residue:

List of descriptors produced by Surface Racer:

1. Curvature in isolation: curvature_isol

2. Curvature in complex: `curvature_complex`

3. Accessible Surface Area (ASA) in isolation: `asa_isol`,

4. Accessible Surface Area (ASA) in complex: `asa_complex`,

5. Molecular Surface Area (MSA) in isolation: `msa_isol`,

6. Molecular Surface Area (MSA) in complex: `msa_complex`,

Density and sponge

The Density descriptor is the sum of total or partial atomic masses of atoms within a probe sphere divided by the volume of such sphere. Following the same kind of approach used to calculate the Density descriptor, but, instead of adding the total or partial mass of the atoms inside the spherical probe, in the Sponge descriptor the volume occupied by each atom is added (using the radius of van der Waals and disregarding the overlap volumes). This volume is then subtracted from and normalized by the volume of the spherical probe, resulting in a measure of the empty space in the nanoenvironment around each residue. Similar to the density calculation, sponge also introduces the same bias for those atoms located on the surface of the molecule. In the same way as the density descriptor, the Blue Star STING has pre-calculated 20 types of variations for Density and Sponge, resulting from the use of a sphere probes with variable radii — from 3 Å to 7 Å, centered on the α-carbons and LHA of each protein residue, and centered at P and C4 atoms of nucleotide residues. In addition, chains in isolation and in complex with other chains present in a given PDB file are considered.

List of Density/Sponge descriptors:

1. Density in isolation for probe sphere radius of 3, 4, 5, 6 and 7 Å centered at Cα:
   `density_CA_3`, `density_CA_4`, `density_CA_5`, `density_CA_6`, `density_CA_7`,

2. Density in isolation for probe sphere radius of 3, 4, 5, 6 and 7 Å centered at LHA: `density_LHA_3`, `density_LHA_4`, `density_LHA_5`, `density_LHA_6`, `density_LHA_7`,

3. Density in complex for probe sphere radius of 3, 4, 5, 6 and 7 Å centered at Cα: `density_CA_3_IFR`, `density_CA_4_IFR`, `density_CA_5_IFR`, `density_CA_6_IFR`, `density_CA_7_IFR`,

4. Density in complex for probe sphere radius of 3, 4, 5, 6 and 7 Å centered at LHA: `density_LHA_3_IFR`, `density_LHA_4_IFR`, `density_LHA_5_IFR`, `density_LHA_6_IFR`, `density_LHA_7_IFR`,

5. Density in isolation for probe sphere radius of 3, 4, 5, 6 and 7 Å centered at P atom of a nucleotide: `density_P_3`, `density_P_4`, `density_P_5`, `density_P_6`, `density_P_7`,

6. Density in isolation for probe sphere radius of 3, 4, 5, 6 and 7 Å centered at C4: `density_C4_3`, `density_C4_4`, `density_C4_5`, `density_C4_6`, `density_C4_7`,

7. Density in complex for probe sphere radius of 3, 4, 5, 6 and 7 Å centered at P atom of a nucleotide: `density_P_3_IFR`, `density_P_4_IFR`, `density_P_5_IFR`, `density_P_6_IFR`, `density_P_7_IFR`,

8. Density in complex for probe sphere radius of 3, 4, 5, 6 and 7 Å centered at C4 atom of a nucleotide: `density_C4_3_IFR`, `density_C4_4_IFR`, `density_C4_5_IFR`, `density_C4_6_IFR`, `density_C4_7_IFR`,

Electrostatic potential

Electrostatic Potential (EP) is calculated using the program Delphi (*6*) according to the modifications done by Walter Rocchia and Goran Neshich (*7*). The EP value is calculated on a

per atom basis and then reported for all eligible PDB format files (including those containing modeled protein structures) in a residue-by-residue fashion. Four pre-calculated categories are stored for each residue:

1. EP at the residue's alpha carbon (CA) atom (`ep_CA`),
2. EP value at the side-chain's last heavy atom (LHA) of amino acid residue (`ep_LHA`),
3. average EP value over all amino acid atoms (`ep_average`), and
4. EP value averaged over the patch of the molecular surface that is attributable to that particular amino acid (`ep_surface`).

It is worth noting that the whole nanoenvironment EP plays a major corrective role in the four final EP reported values.

Entropy density

The Entropy Density descriptor is similar to the Contact Energy Density descriptor, but instead of the summing contacts energy values within a probe sphere, we use the relative entropy calculated according to HSSP (*8*). Then the sum of relative entropies is divided by the volume of the probe sphere. Entropy in this case is referred to as disorder observable at certain location in alignment of homologous primary sequences. The Entropy Density is calculated by centering the probe sphere at the C-α and LHA atoms of each residue, and considering both the protein chain in isolation and in complex.

List of Entropy Density descriptors:

1. Probe sphere of radii 3, 4, 5, 6 and 7 Å centered at C-α for protein chain in isolation: `entd_CA_3`, `entd_CA_4`, `entd_CA_5`, `entd_CA_6`, `entd_CA_7`,

2. Probe sphere of radii 3, 4, 5, 6 and 7 Å centered at LHA for protein chain in isolation: `entd_LHA_3`, `entd_LHA_4`, `entd_LHA_5`, `entd_LHA_6`, `entd_LHA_7`,

3. Probe sphere of radii 3, 4, 5, 6 and 7 Å centered at C-α for protein chain in complex: `entd_CA_3_IFR`, `entd_CA_4_IFR`, `entd_CA_5_IFR`, `entd_CA_6_IFR`, `entd_CA_7_IFR`,

4. Probe sphere of radii 3, 4, 5, 6 and 7 Å centered at LHA for protein chain in complex: `entd_LHA_3_IFR`, `entd_LHA_4_IFR`, `entd_LHA_5_IFR`, `entd_LHA_6_IFR`, `entd_LHA_7_IFR`,

Graph descriptor

Protein chains can be represented as undirected graphs where the set of vertices is composed of a protein chain's amino acid residues (or atoms), while the edges of the graph represent interactions between these residues (or atoms) (*9–12*). In Blue Star STING, the amino acid residues of a protein were used as a set of vertices, and the set of edges is defined using previously calculated interatomic contacts. From a graph, it is possible to obtain several metrics and measures that extract and describe the behavior of protein chains as networks of interactions between amino acid residues. Representing geometric and topological properties of protein chains, these metrics can be considered as structural descriptors of proteins, since the graphs are constructed based on structural information of the protein chains.

List of Graph descriptors (for a definition of the graph metrics see (*13–15*)):

1. Eccentricity: (`eccentricity`),

2. Radiality Centrality: `radiality_centrality`,

3. Local Closeness: `local_closeness`,

4. Dice similarity: `dice_similarity`,
5. Mean Neighbor Degree (MND): `mean_neighbor_degree`,
6. Local average centrality (LAC): `lac`,
7. Density of Maximum Neighborhood Component (DMNC): `dmnc`,
8. Closeness: `closeness`,
9. Cluster coefficient: `cluster_coefficient`,
10. Degree: `degree`,
11. Betweenness: `betweenness`,
12. Random walk betweenness: `random_walk_betweenness`,
13. Bary center: `bary_center`,
14. Page rank: `page_rank`,
15. Bottleneck: `bottle_necck`

Hydrophobicity

Blue Star STING calculates hydrophobicity using the hydrophobicity scales defined by RADZICKA & WOLFENDEN (*16*) and KYTE & DOOLITTLE (*17*). The Hydrophobicity of an amino acid residue i (where i is the residue sequence position in the protein's primary structure), of type t, is calculated using the value stipulated in above mentioned scales, weighted by the relative accessibility to the solvent (acc_{max}). We calculate hydrophobicity for each residue using the accessibilities for the protein chain in isolation and in complex.

$$Hydrophobicity_i = \frac{acc_i}{acc^t_{max}} * Hydrophobicity^S_t \tag{4}$$

where acc_i is the accessibility of residue i (in isolaiton or in complex), acc^t_{max} is the absoluute accessibility for the residue of type t and $Hydrophobicity^S_t$ is the hydrophobicity for the residue of type t as defined in the scale S.

List of hydrophobicity descriptors:

1. Hydrophobicity in isolation using the RADZICKA & WOLFENDEN scale: `hydro_radzicka_isol_surfv`, `hydro_radzicka_isol_naccess` and `hydro_radzicka_isol_nsc`,

2. Hydrophobicity in complex using the RADZICKA & WOLFENDEN scale: `hydro_radzicka_complex_surfv`, `hydro_radzicka_complex_naccess` and `hydro_radzicka_complex_nsc`,

3. Hydrophobicity in isolation using the KYTE & DOOLITTLE scale: `hydro_kite_dolitte_isol_surfv`, `hydro_kite_dolitte_isol_naccess` and `hydro_kite_dolitte_isol_nsc`,

4. Hydrophobicity in complex using the KYTE & DOOLITTLE scale: `hydro_kite_dolitte_complex_surfv`, `hydro_kite_dolitte_complex_naccess` and `hydro_kite_dolitte_complex_nsc`,

Residue contacts

Amino acid contacts in terms of atomic interactions are essential factors to be considered in the analysis of a protein's structure and its complexes. Residue-residue contacts are calculated according to description given in Mancini et al. (*5*). Contact types considered in STING RDB are:

1. Hydrophobic interactions (energy: $0.6\,\mathrm{Kcal/mol}$),

2. Hydrogen Bonding (energy: $2.6\,\mathrm{Kcal/mol}$),
3. Aromatic Stacking (energy: $1.5\,\mathrm{Kcal/mol}$),
4. Salt bridging (energy: $10.0\,\mathrm{Kcal/mol}$),
5. Cysteine-bridging (energy: $85.0\,\mathrm{Kcal/mol}$).

Secondary structure

In Blue Star STING, there are three different secondary structure assignments for an amino acid residue, obtained by consulting information contained in the PDB files themselves (when available) and those calculated by softwares DSSP (*18*) and STRIDE (*19*). Often, the types of secondary structures, as well as the initial and final amino acid position in addition to sizes of particular secondary structure elements (SSE), diverge between the different classifications. Thus, with the presence of values/descriptions in STING RDB coming from three different sources, it is possible to obtain a consensus and find regions with more reliable secondary structures assignments. Noteworthy is the observation that some protein districts appear to have preferences to specific secondary structure configuration in order to perform certain function (structural or enzymatic). Each program (DSSP and STRIDE) produces different outputs, which we store in STING RDB2. In order to make them comparable we provide a mapping between the program's secondary structure encoding schema to a common schema: **H** (alpha helix); **G** (310 helix); **I** (Pi Helix); **E** or **D** (extended strand in parallel and/or anti-parallel B-sheet conformation); **B** or **b** (isolated B-bridge); **T** (turn); **C** (coil); **S** (bend).

List of descriptors produced by DSSP:

1. Secondary structure: one of the codes in the common schema (`secondary_structure`),
2. Kappa: virtual bond angle (bend angle) defined by the three Cα atoms of residues $i-2$, i, $i+2$. Used to define bend (structure code **S**) (`kappa`),

3. Dihedral angles ϕ and ψ (`psi` and `psi`),

4. Accessibility: DSSP calculated accessibility (`accessibility`)

List of descriptors produced by STRIDE:

1. Secondary structure: one of the codes in the common schema (`secondary_structure`),

2. Secondary structure elements: delimitation of starting and ending residues of a SS element (consecutive residues forming a major SS) - first residue in a SS element (>), last residue in a SS element (<) and a residue within a SS element (=),

3. Dihedral angles ϕ and ψ (`psi` and `psi`),

4. Accessibility: DSSP calculated accessibility (`accessibility`)

Side chain orientation

The side chain orientation (*20*) is calculated for each amino acid residue in a protein chain as an angle formed between two vectors: Cα-CENTROID and Cα-LHA. The Cα-CENTROID is the vector from the C-α atom of an amino acid residue to the center of mass of a specific region (probe sphere), and the Cα-LHA is the vector from the C-α atom to the Last Heavy Atom of that same amino acid residue. Then, for each amino acid residue, we calculate how much their side chains (Cα-LHA vector) deviate from the vector pointing to the center of mass of the probing sphere. Additionally, we also calculate the average angle of all amino acid residues found within the probing sphere. Finally, we subtract that angle calculated for each amino acid separately, from the average one, giving us a description of how divergent or convergent the side chain of any residue is compared to its neighbors.

List of Side Chain Orientation descriptors:

1. Side chain orientation angle for probe sphere of radii 3, 4, 5, 6, and 7 Å: `side_chain_angle_3`, `side_chain_angle_4`, `side_chain_angle_5`, `side_chain_angle_6`, `side_chain_angle_7`,

2. Side chain orientation using average angle for probe sphere of radii 3, 4, 5, 6, and 7 Å: `side_chain_average_angle_3`, `side_chain_average_angle_4`, `side_chain_average_angle_5`, `side_chain_average_angle_6`, `side_chain_average_angle_7`,

3. Side chain orientation angle of neighbor atoms for probe sphere of radii 3, 4, 5, 6, and 7 Å: `neighbors_side_chain_angle_3`, `neighbors_side_chain_angle_4`, `neighbors_side_chain_angle_5`, `neighbors_side_chain_angle_6`, `neighbors_side_chain_angle_7`

Solvation (energy)

The solvation energy corresponds to the energy of atom bonds established between the solute and the solvent. In case the solvent is water, this is also called the free energy of hydration. From the relative solvent accessible area of each protein atom, it is possible to calculate approximately the free energy of hydration that originates from the interactions between such atoms and the water molecules (solvent) (*21*)). The free energy of hydration of the i-th atom of an amino acid residue is calculated as the product of its experimentally determined atomic solvation parameter (g_i) and the relative solvent accessible area (RSA). The sum of the free energies of hydration of the atoms that make up the residue r and the atoms in the vicinity of r, normalized by the sum of the relative areas accessible to the solvent of all atoms considered, gives the solvation energy of the residue r (G_r) with the formula:

$$G_r = \frac{\sum_i g_i ASA_{relative}}{\sum_i ASA_{relative}} \quad (5)$$

List of Solvation descriptors:

1. Solvation for pobre sphere of radii 3, 4, 5, 6, and 7 Å: `solvation_3`, `solvation_4`, `solvation_5`, `solvation_6`, `solvation_7`

Unused contacts

Each residue can make certain (maximum) number of contacts. The difference between the maximum number of contacts and the contacts established is defined as "unused contacts". We maintain a table describing the maximum number of interatomic contacts identified for each of the 20 amino acid types (`http://www.cbi.cnptia.embrapa.br/SMS/STINGm/help/table_of_max_number_contacts.html`). The contacts are classified by the contact type (and residue type) and were extracted only from those PDB files containing the structures resolved by the X-ray crystallography and having the resolution better or equal to 2.0 Å. Furthermore, structures with identified double occupancy atoms, were not considered. This table is consulted with every PDB update and if necessary, numbers in the table are updated to reflect possible changes in occurrence of maximum number of contacts for each contact type and each residue type. When the table is updated the Unused Contacts descriptor is recalculated for all structures in the PDB.

List of Unused Contacts descriptors:

1. Number of unused contacts per contact type:
    - Hydrophobic: `hydrophobic_uc`,
    - Charge attractive: `charge_attr_uc`,
    - Charge repulsive: `charge_repu_uc`,

- H-Bond between atoms of the residues' main chains, including zero, one or two intermediate waters: hb_mm_uc, hb_mwm_uc and hb_mwwm_uc,
- H-Bond between atoms of the residues' side chains, including zero, one or two intermediate waters: hb_ss_uc, hb_sws_uc and hb_swws_uc,
- H-Bond between atoms of the residues' main chain and side chain , including zero, one or two intermediate waters: hb_ms_uc, hb_mws_uc and hb_mwws_uc,
- Aromatic: aromatic_uc,
- Disulfide bridge: ss_bond_uc

2. Energy of unused contacts per contact type:

- Hydrophobic: hydrophobic_uc_energy,
- Charge attractive: charge_attr_uc_energy,
- Charge repulsive: charge_repu_uc_energy,
- H-Bond between atoms of the residues' main chains, including zero, one or two intermediate waters: hb_mm_uc_energy, hb_mwm_uc_energy and hb_mwwm_uc_energy,
- H-Bond between atoms of the residues' side chains, including zero, one or two intermediate waters: hb_ss_uc_energy, hb_sws_uc_energy and hb_swws_uc_energy,
- H-Bond between atoms of the residues' main chain and side chain , including zero, one or two intermediate waters: hb_ms_uc_energy, hb_mws_uc_energy and hb_mwws_uc_energy,
- Aromatic: aromatic_uc_energy,
- Disulfide bridge: ss_bond_uc_energy

Weighted contact number

The Weighted Contact Number (WCN) is a measure of backbone flexibility of amino acid residues (*20*). For each amino acid residue in the protein chain we calculated the WCN and average WCN according to the following equations:

$$WCN_i = \sum_{j \neq i} \frac{1}{r_{ij}^2} \tag{6}$$

where j is any other residues in the protein chain and r_{ij}^2 is the squared distance between the Cα atoms of residue i and j, and

$$\overline{WCN_i} = \sum_{j \in k} \frac{z_j}{K} \tag{7}$$

where k are the nearest neighbors of residue i (square euclidean distance between Cα atoms), z_j is the normalized WCN of residue j (z-score) and K is the number of nearest neighbors residue ($|k| \leq K$).

List of Weighted Contact Number descriptors:

1. Weighted contact number: `weighted_contact_number`,
2. Average Weighted contact number for layers (k) of 2, 3, 4 and 5: `avg_weighted_contact_number_k_2`, `avg_weighted_contact_number_k_3`, `avg_weighted_contact_number_k_4`, `avg_weighted_contact_number_k_5`

Neighbor descriptors

In addition to the previously described descriptors, we calculated a class of descriptors called *Neighbor Descriptors*. The Neighbor Descriptors (ND) are calculated by performing an aggregation of a *base descriptor* using a neighborhood definition.

Weighted neighbor average descriptor

This descriptor is a type of a Neighbor Descriptor (ND) inspired by the work of POROLLO & MELLER (*22*). The Weighted Neighbor Average or simply WNA were calculated and stored. There are two Weighted Neighbor Average (WNA) descriptors for each *base descriptor* in Blue Star STING. The first of them uses values of the relative accessibility (RSA) for the neighboring residues used for adding specific weight to the base descriptors (therefore yielding the WNA-Surface), and the second one uses the inverse of the distance between the central residue and its neighbors (WNADistance). To define the neighborhood for a selected residue, a sphere of $15\,\text{Å}$ radius centered on residue's α-carbon was used. In the case of WNASurface descriptors that use relative accessibility as a weighting factor, only residues with a relative area accessible to the solvent greater than 5% are considered. The neighbor descriptors are calculated for all numerical descriptors in Blue Star STING.

For each *base descriptor* d_i of a residue i the WNASurface descriptor is calculated as follows:

$$WNASurface_i = \frac{\sum_{j \in N_i \wedge acc\_rsa_j > 5\%} acc\_rsa_j * d_j}{V(r)} \tag{8}$$

where N_i is the set of neighbor residues of the residue i (including i itself), acc_rsa_j is the RSA of residue j, d_j is the value of the *base descriptor* for residue j and $V(r)$ is the volume of the sphere (i.e., the neighborhood). In this work we use $r = 15\,\text{Å}$.

The WNADistance descriptor is calculate as:

$$WNADistance_i = \frac{\sum_{j \in N_i} \frac{d_j}{D_{i,j}}}{V(r)} \tag{9}$$

where $D_{i,j}$ is the euclidean distance between residues i and j.

Sliding window neighbor descriptor

The Sliding Window (SW) uses the primary structure to define the neighborhood of a residue. For each *base descriptor* d_i of a residue i the SW descriptor is calculated as follow:

$$SW_i = \frac{\sum_{(i-L/2)\leq j\leq (i+L/2)} d_i}{L} \tag{10}$$

where L is the window length and j is a sequence neighbor of residue i.

STING RDB2 stores SW descriptors calculated using four window lengths: 3, 5, 7, and 9 amino acids. The SW descriptors are calculated for all numerical descriptors and they are named in the form `base_descriptor_name_SW_`L.

Graph neighbor descriptor

In the same way as the graphs are constructed to represent protein chains, they can be used to define a neighborhood. The neighborhood can then be used for calculating neighbor descriptors.

For this, a maximum value is defined for the neighborhood size (K), representing the number of edges or the length of the shortest path between a central residue and its neighbors. That is, for $K = 1$, only the vertices immediately adjacent to the central atom are considered as neighbors. In the case of $K = 2$, the adjacent neighbors plus the neighbors of these neighbors are considered, and so on for larger values of K. Therefore, let r_i be any amino acid residue and f_i be a *base descriptor* for this residue, then the Graph Neighbor (GN) descriptor can be calculated for the neighborhood (g_{f_i}) from the graph $G(V, E)$, where V is the set of vertices or amino acid residues and E is the set of edges or contacts. Considering as "neighbors" of the vertex r_i, those amino acid residues that were visited by a minimum path of a maximum length (equal to K), where $d_{i,j}$ is the distance (number of edges) between the residue and its neighbor j, the calculation of g_{f_i} is performed considering different weights for the neighboring layers

($k = 1, 2, 3, ..., K$):

$$g_{f_i} = f_i + \sum_{k=1}^{K} \frac{\sum_{\forall j | d(i,j) < k} f_j}{k^2} \quad (11)$$

The STING RDB2 stores pre-calculated GN descriptor using $K = 6$, and the descriptors are named in the form: `base_descriptor_name_GN`.

All descriptors

For all descriptors used in this work, we used STING SDLg (Sting Data Library generator) to calculate them in all possible variants (meaning, using all values for variables used to calculate each one of them) and to apply batch calculations on sgRNA-DNA-Cas9 complexes modeled in the molecular dynamics simulations as described previously.

1 Supplementary Methods

1.1 SHapley Additive exPlanations

Inspired by coalition game theory, SHapley Additive exPlanations (SHAP) computes the contribution of individual features towards the XGBoost model's predictions. SHAP does this for a given datapoint by assigning SHAP values to each input feature such that the SHAP values sums to the model's prediction minus a constant baseline. Mathematically, for datapoint i:

$$m(X^{(i)}) = \hat{y}^{(i)} = t(X^{(i)}) = b + \sum_{j=1}^{|F|} \phi_j^{(i)} \quad (12)$$

where:

- m is the ML model,
- t is the explanation model,
- F is the set of input features to m,

- $\hat{y}^{(i)} \in \mathbb{R}$ is the predicted CRISPR-Cas9 cleavage activity value for datapoint i,
- $X^{(i)} \in \mathbb{R}^{|F|}$ is the input feature vector for datapoint i,
- $b \in \mathbb{R}$ is some constant baseline, and
- $\phi_j^{(i)}$ the SHAP value assigned to feature j for datapoint i.

SHAP values can be used for both local or global interpretation. While local interpretation allows one to explain individual predictions, we are more interested in global interpretation. Specifically, global interpretation allow us to quantify and rank the importance of input features. Namely, from SHAP values ϕ, the SHAP importance I_j of a feature j in a model m can be quantified by the following equation:

$$I_j = \frac{1}{N} \sum_{i=1}^{N} |\phi_j^{(i)}| \tag{13}$$

where N is the number of datapoints. Similarly, the SHAP importance I_J of a feature group J (e.g., a set of features sharing a common CRISPR-Cas9 residue or STING descriptor class) can be quantified by the following equation:

$$I_J = \frac{1}{N} \sum_{i=1}^{N} \Big| \sum_{j \in J} \phi_j^{(i)} \Big| \tag{14}$$

SHAP values also allows us to identify how variations in the feature value for a single feature impacts the model's output across the whole dataset. This allows us to study how changes in input feature values affect model predictions within the dataset.

The Python SHAP package provides an API for producing SHAP summary plots from SHAP values. Normally limited to 20 rows, each row in a SHAP summary plot is a horizontal beeswarm plot for each input feature, where the input features are ordered by decreasing SHAP feature importance. Dots in the beeswarm plot for each feature are colored by the datapoint's feature value. Red, purple and blue dots in the plot correspond to high, medium and low feature values, respectively.

1.2 Model interpretation

We compute feature counts and SHAP importances of parent descriptor classes for each residue cluster by stratifying features into their respective residue clusters.

Cas9 residues may be on the surface of the isolated protein, the surface of the Cas9 complex, and/or the interface between Cas9 and non-Cas9 components in the complex. However, unlike the previous properties, these properties dynamically change across different PDB snapshots. For example, a residue may be on the interface in some snapshots but not in others. Based on the above, we compute:

- the average number of surface residues $\frac{1}{|D|}\sum_{i=1}^{|D|}\alpha_r^{(i)}$ and non-surface residues $\frac{1}{|D|}\sum_{i=1}^{|D|}(1-\alpha_r^{(i)})$
- the SHAP importance of surface residues $I_{\text{surface}} = \frac{1}{|D|}|\sum_{r\in R}(\alpha_r^{(i)}\sum_{j\in F(r)}\phi_j^{(i)})|$ and non-surface residues $I_{\text{non-surface}} = \frac{1}{|D|}|\sum_{r\in R}((1-\alpha_r^{(i)})\sum_{j\in F(r)}\phi_j^{(i)})|$
- the average number of interface residues $\frac{1}{|D|}\sum_{i=1}^{|D|}\alpha_{r,IFR}^{(i)}$ and non-interface residues $\frac{1}{|D|}\sum_{i=1}^{|D|}(1-\alpha_{r,IFR}^{(i)})$; and
- the SHAP importance of interface residues $I_{\text{interface}} = \frac{1}{|D|}\sum_{r\in R}(\alpha_{r,IFR}^{(i)}\sum_{j\in F(r)}\phi_j^{(i)})|$ and non-interface residues $I_{\text{non-surface}} = \frac{1}{|D|}|\sum_{r\in R}((1-\alpha_{r,IFR}^{(i)})\sum_{j\in F(r)}\phi_j^{(i)})|$

where:

- R is the set of residues in STING_CRISPR;
- $F(r)$ is the set of features with residue r;
- $\alpha_r^{(i)} = 1$ if residue r is a surface residue in PDB snapshot i, and 0 otherwise; and
- $\alpha_{r,IFR}^{(i)} = 1$ if residue r is an interface residue in PDB snapshot i, and 0 otherwise.

We also compute the fraction of snapshots in which a residue is a surface or an interface residue, which are given by $\frac{1}{|D|}\sum_{i=1}^{|D|}\alpha_r^{(i)}$ and $\frac{1}{|D|}\sum_{i=1}^{|D|}\alpha_{r,IFR}^{(i)}$, respectively. We repeat the above calculations for the three accessibility tools SurfV (*1*), NACCESS (*3*) and NSC (*4*). We use bar plots to visualize the above SHAP importances, and use a heatmap to visualize the fractions.

To quantify the importance of HPR for predicting CRISPR-Cas9 cleavage activity, we repeat the same training procedure, but considered the $1361\dot{1}671 = 2274231$ features spanning Cas9 residues $3 - 1363$ in place of the 380 HPRs, thereby obtaining a different model STING_CRISPR_ALL. HPRs are not always proximal to the heteroduplex in all PDB snapshots. Because of this, using STING_CRISPR_ALL, we calculate:

- feature counts of HPRs$\frac{1}{|D|}\sum_{i=1}^{|D|}\delta_{r,HPR}^{(i)}$ and non-HPRs $\frac{1}{|D|}\sum_{i=1}^{|D|}(1-\delta_{r,HPR}^{(i)})$;
- SHAP importance of HPRs $\frac{1}{|D|}\sum_{i=1}^{|D|}|\sum_{r\in R}\delta_{r,HPR}^{(i)}(\sum_{j\in F(r)}\phi_j^{(i)})|$; and
- SHAP importance of non-HPRs $\frac{1}{|D|}\sum_{i=1}^{|D|}|\sum_{r\in R}(1-\delta_{r,HPR}^{(i)})(\sum_{j\in F(r)}\phi_j^{(i)})|$,

where $\delta_{r,HPR}^{(i)} = 1$ if residue r is a HPR in PDB snapshot i, and 0 otherwise, and $F(r)$ is the set of features with residue r, and use heatmaps to visualize the importances.

We also project residue importances onto the heteroduplex bases to measure their importances. Namely, for a given heteroduplex base b, we can quantify its importance I_b by the following equation: $I_b = \sum_{i=1}^{|D|}|\sum_{r\in R(b)}d_r^{(i)}(\sum_{j\in F(r)}\phi_j^{(i)})|$, where $R(b)$ denotes the set of residues whose α-carbon atom is 3-7 Å away from the C4' atom of base b. Similarly, we can also quantify the SHAP importance of residue-base pairs by calculating $I_{b,r} = \sum_{i=1}^{|D|}|d_r^{(i)}(\sum_{j\in F(r)}\phi_j^{(i)})|$.

1.3 Raw data

Target Type	CMUT	Target Site (Non-Target Strand)	Mutation	CRISPR-Cas9 Cleavage Activity
On-target	CMUT1	GACGCATAAAGATGAGACGCTGG	None	0.0928059068718
Off-target	CMUT2	G**C**CGCATAAAGATGAGACGCTGG	A19C	0.0787201649863
	CMUT8	G**G**CGCATAAAGATGAGACGCTGG	A19G	0.181542289862
	CMUT4	G**T**CGCATAAAGATGAGACGCTGG	A19T	0.114618189415
	CMUT5	GA**A**GCATAAAGATGAGACGCTGG	C18A	0.132345410767
	CMUT3	GA**G**GCATAAAGATGAGACGCTGG	C18G	0.119446527474
	CMUT7	GA**T**GCATAAAGATGAGACGCTGG	C18T	0.14436947128
	CMUT10	GAC**A**CATAAAGATGAGACGCTGG	G17A	0.115995295517
	CMUT13	GAC**C**CATAAAGATGAGACGCTGG	G17C	0.106781628351
	CMUT19	GAC**T**CATAAAGATGAGACGCTGG	G17T	0.0932489445754
	CMUT21	GACG**A**ATAAAGATGAGACGCTGG	C16A	0.0196421394474
	CMUT9	GACG**G**ATAAAGATGAGACGCTGG	C16G	0.00859648371669
	CMUT12	GACG**T**ATAAAGATGAGACGCTGG	C16T	0.111148283845
	CMUT29	GACGC**C**TAAAGATGAGACGCTGG	A15C	0.0945242769362
	CMUT23	GACGC**G**TAAAGATGAGACGCTGG	A15G	0.000971813092723
	CMUT27	GACGC**T**TAAAGATGAGACGCTGG	A15T	0.00493182712775
	CMUT17	GACGCA**A**AAAGATGAGACGCTGG	T14A	0.00208172468785
	CMUT30	GACGCA**C**AAAGATGAGACGCTGG	T14C	0.0235738673331
	CMUT28	GACGCA**G**AAAGATGAGACGCTGG	T14G	0.000458729016148
	CMUT25	GACGCAT**C**AAGATGAGACGCTGG	A13C	0.00116725554782
	CMUT20	GACGCAT**G**AAGATGAGACGCTGG	A13G	0.00389589061704
	CMUT26	GACGCAT**T**AAGATGAGACGCTGG	A13T	0.0111729840733
	CMUT18	GACGCATA**C**AGATGAGACGCTGG	A12C	0.00228748213652
	CMUT14	GACGCATA**G**AGATGAGACGCTGG	A12G	0.0201204142155
	CMUT24	GACGCATA**T**AGATGAGACGCTGG	A12T	0.0815231184044
	CMUT22	GACGCATAA**C**GATGAGACGCTGG	A11C	0.00465650328521
	CMUT16	GACGCATAA**G**GATGAGACGCTGG	A11G	0.00586891296755
	CMUT11	GACGCATAA**T**GATGAGACGCTGG	A11T	0.0561840566924
Off-target (unused)	CMUT6	**A**ACGCATAAAGATGAGACGCTGG	G20A	0.0702299155412
	CMUT15	**T**ACGCATAAAGATGAGACGCTGG	G20T	0.191606618035

Table S1: The 30 (1 on-target and 29 off-target) CRISPR-Cas9 guide-target interfaces initially considered in this study. Sorted by mismatch position, 27 of the 29 off-target interfaces consist of single mismatches +19 to +11 nucleotides away from the PAM. Cleavage activity values are extracted from the column "wtCas9_cleave_rate_log" within Supplementary File 2 of Jones Jr. et al. (*23*). All-atom molecular dynamic trajectories are not produced for CMUT6 and CMUT15, and thus they are discarded when creating the machine learning dataset.

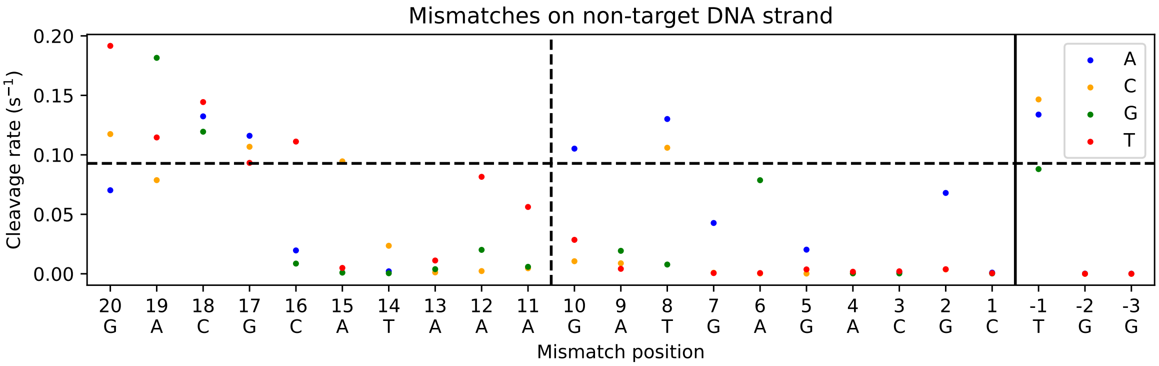

Figure S1: CRISPR-Cas9 (off-)target cleavage activity for on- and off-target interfaces listed in Table S1, ultimately from Jone Jr. et al. (*23*). The nucleotide sequence under the x-axis shows the nucleotides in the on-target interface's non-target strand, and the dots' colors show the resulting mutated nucleotide at the particular nucleotide position. The horizontal dotted line shows the on-target's activity, and the vertical dotted line separates PAM-distal positions (+20 to +11) from PAM-proximal positions (+10 to +1).

1.4 Heteroduplex-proximal residues

This study considers six sets of sgRNA-tsDNA heteroduplex-proximal residues (HPRs): one for each fold during five-fold cross validation and one for the train-test split. In summary, all six HPR sets share the following Cas9 residues: 13, 56, 57, 58, 59, 60, 61, 62, 63, 64, 65, 66, 67, 68, 69, 70, 71, 72, 73, 74, 75, 78, 136, 139, 160, 161, 162, 163, 164, 165, 166, 167, 168, 169, 170, 171, 218, 249, 260, 263, 265, 266, 267, 268, 269, 270, 271, 301, 317, 321, 324, 362, 364, 365, 366, 367, 368, 369, 370, 371, 374, 396, 400, 401, 402, 403, 404, 405, 406, 407, 408, 409, 410, 411, 412, 414, 415, 443, 444, 445, 446, 447, 448, 449, 450, 451, 452, 453, 454, 455, 456, 460, 461, 462, 463, 464, 465, 475, 478, 488, 489, 491, 492, 493, 494, 495, 496, 497, 498, 499, 500, 501, 503, 506, 507, 508, 509, 510, 511, 515, 516, 518, 519, 520, 521, 522, 523, 524, 525, 526, 527, 528, 529, 536, 537, 538, 539, 557, 558, 559, 560, 561, 580, 581, 582, 583, 584, 585, 586, 587, 588, 589, 590, 591, 592, 624, 625, 626, 627, 628, 631, 655, 656, 657, 658, 659, 660, 661, 662, 663, 666, 667, 683, 688, 689, 690, 691, 692, 693, 694, 695, 696, 697, 698, 699, 700, 701, 702, 705, 708, 709, 711, 712, 713, 714, 715, 716, 717, 718, 719, 720, 721, 722, 723, 724, 725, 726, 727, 728, 729, 730, 731, 732, 733, 734, 737, 761, 763, 764, 765, 766, 767, 768, 769, 770, 771, 772, 773, 774, 775, 776, 777, 778, 779, 780, 781, 782, 783, 784, 802, 803, 804, 806, 807, 808, 809, 810, 812, 816, 833, 834, 835, 836, 837, 838, 839, 840, 842, 843, 844, 845, 846, 847, 848, 849, 850, 851, 852, 853, 854, 855, 859, 860, 861, 862, 864, 866, 867, 868, 869, 893, 895, 896, 908, 913, 916, 917, 918, 919, 920, 921, 922, 923, 924, 925, 926, 927, 928, 929, 930, 931, 932, 933, 936, 937, 941, 948, 949, 951, 955, 956, 957, 958, 959, 960, 961, 1003, 1004, 1005, 1006, 1007, 1008, 1009, 1010, 1011, 1012, 1013, 1014, 1015, 1016, 1017, 1018, 1019, 1020, 1021, 1022, 1023, 1024, 1025, 1026, 1027, 1028, 1029, 1030, 1031, 1032, 1033, 1034, 1035, 1036, 1038, 1039, 1106, 1107, 1108, 1109, 1110, 1111, 1122, 1134, 1135, 1136, 1138. Each HPR set additionally have the following residues:

- Fold 0 (15 extra residues): 261, 416, 512, 621, 629, 710, 762, 813, 858, 863, 870, 910, 934, 938, 940;
- Fold 1 (16 extra residues): 217, 261, 416, 490, 512, 621, 629, 710, 762, 813, 858, 863, 870, 910, 934, 940;
- Fold 2 (13 extra residues): 217, 261, 416, 490, 621, 629, 710, 762, 813, 858, 870, 910, 938;
- Fold 3 (13 extra residues): 217, 261, 416, 490, 512, 629, 762, 858, 863, 910, 934, 938, 940;
- Fold 4 (11 extra residues): 217, 490, 512, 621, 710, 813, 863, 870, 934, 938, 940
- Train-test split (17 extra residues): 217, 261, 416, 490, 512, 621, 629, 710, 762, 813, 858, 863, 870, 910, 934, 938, 940

In terms of counts, HPRs for the five folds have 378, 379, 376, 376 and 374 residues, respectively, and the train-test split HPR has 380 residues.

1.5 STING_CRISPR: an ExtraTrees model with Cas9 STING features

Table S3 lists all 21 features grouped by STING descriptors. In total, there are 9 unique descriptor classes, 16 unique descriptors and 15 unique amino acids (136, 271, 317, 406, 730, 731, 732, 733, 734, 837, 838, 839, 925, 1015, 1016) among the amino acid-specific STING descriptor features.

Parent descriptor class (neighbor aggregations)	No. of descriptors	No. of features
Accessibility	15	5700
Cross Link Order (GN, SW, WNA, VD)	216 $(= 27 + 108 + 54 + 27)$	82080
Cross Presence Order (GN, SW, WNA, VD)	216 $(= 27 + 108 + 54 + 27)$	82080
Curvature (GN, SW, WNA, VD)	96 $(= 12 + 48 + 24 + 12)$	36480
Density (GN, SW, WNA, VD)	160 $(= 20 + 80 + 40 + 20)$	60800
Sponge (GN, SW, WNA, VD)	160 $(= 20 + 80 + 40 + 20)$	60800
Contact Energy Density (GN, SW, WNA, VD)	160 $(= 20 + 80 + 40 + 20)$	60800
DSSP	15	5700
Stride	13	4940
Electrostatic Potential (GN, SW, WNA, VD)	32 $(= 4 + 16 + 8 + 4)$	12160
Entropy Density (GN, SW, WNA, VD)	160 $(= 20 + 80 + 40 + 20)$	60800
Graph Descriptor (GN, SW, WNA, VD)	128 $(= 16 + 64 + 32 + 16)$	48640
Hydrophobicity	12	4656
Residue Contact (GN, SW, WNA, VD)	72 $(= 9 + 36 + 18 + 9)$	27360
Side Chain Orientation (GN, SW, WNA, VD)	120 $(= 15 + 60 + 30 + 15)$	45600
Solvation (GN, SW, WNA, VD)	40 $(= 5 + 20 + 10 + 5)$	15200
Unused Contacts (GN, SW, WNA, VD)	16 $(= 2 + 8 + 4 + 2)$	6080
Weighted Contact Number (GN, SW, WNA, VD)	40 $(= 5 + 20 + 10 + 5)$	15200
Total	1671	634980

Table S2: Number of descriptors and features generated from the 60 STING descriptor classes used for characterizing CRISPR-Cas9's internal protein nanoenvironment in this study. The left column lists the 17 parent descriptor classes and their corresponding list of relevant neighbor aggregation methods (GN = Graph Neighbors, SW = Sliding Window, WNA = Weighted Neighbor Average, VD = Voronoi Diagram). The middle column shows the total number of descriptors for each parent descriptor class, with a breakdown of the count enclosed in parentheses if neighbor descriptors are used instead. The right column shows the number of residue-resolved features considered as ML input features for each parent descriptor class when building STING_CRISPR. For STING_CRISPR, we use 380 sgRNA-target strand DNA heteroduplex-proximal residues in order to model the CRISPR-Cas9 protein 3D nanoenvironment close to the sgRNA-target strand DNA heteroduplex. As a result, numbers on the right column is calculated by multiplying the number of descriptors by 380, the number of heteroduplex-proximal residues. In total, there are 634980 amino acid-resolved features considered in this study.

Parent descriptor class	Neighbour Type	Descriptor name	CRISPR-Cas9 residue(s)
Accessibility	-	acc_ifr_surfv	730
Accessibility	-	acc_isol_surfv	837
Contact Energy Density	VD	ced_CA_4_VD	837
Contact Energy Density	WNA	ced_LHA_4_WNADist	164
Cross Presence Order	SW	cpo_85_30_CB_SW_3	730
Cross Presence Order	WNA	cpo_85_30_CB_WNADist	136
Cross Presence Order	WNA	cpo_85_15_CB_WNADist	908
Density	GN	density_CA_7_IFR_GN	415
Density	SW	density_LHA_6_SW_3	408
Density	SW	density_LHA_7_SW_7	734
Density	SW	density_CA_4_SW_3	919
Density	SW	density_LHA_6_SW_3	1017
Density	VD	density_LHA_5_IFR_VD	402
Density	VD	density_CA_7_VD	732
Electrostatic Potential	GN	ep_average_GN	317
Electrostatic Potential	WNA	ep_average_WNADist	839
Entropy Density	SW	entd_CA_5_SW_5	268
Entropy Density	VD	entd_CA_5_VD	837
Entropy Density	WNA	entd_CA_3_WNADist	415
Entropy Density	WNA	entd_CA_6_WNADist	838
Graph Descriptor	SW	betweenness_SW_7	1122
Graph Descriptor	VD	closeness_VD	728
Side Chain Orientation	SW	side_chain_angle_3_SW_7	411
Side Chain Orientation	VD	neighbors_side_chain _angle_3_VD	733
Solvation	VD	solvation_4_VD	1010
Sponge	SW	sponge_CA_6_IFR_SW_5	408
Sponge	SW	sponge_CA_7_SW_5	1016
Weighted Contact Number	GN	avg_weighted_contact _number_k_5_GN	732
Weighted Contact Number	SW	weighted_contact _number_SW_9	733
Weighted Contact Number	SW	avg_weighted_contact _number_k_3_SW_9	1025

Table S3: The 30 input features used in STING_CRISPR. Features are grouped by descriptor classes (in alphabetical order), and subsequently sorted in ascending CRISPR-Cas9 residue numbers.

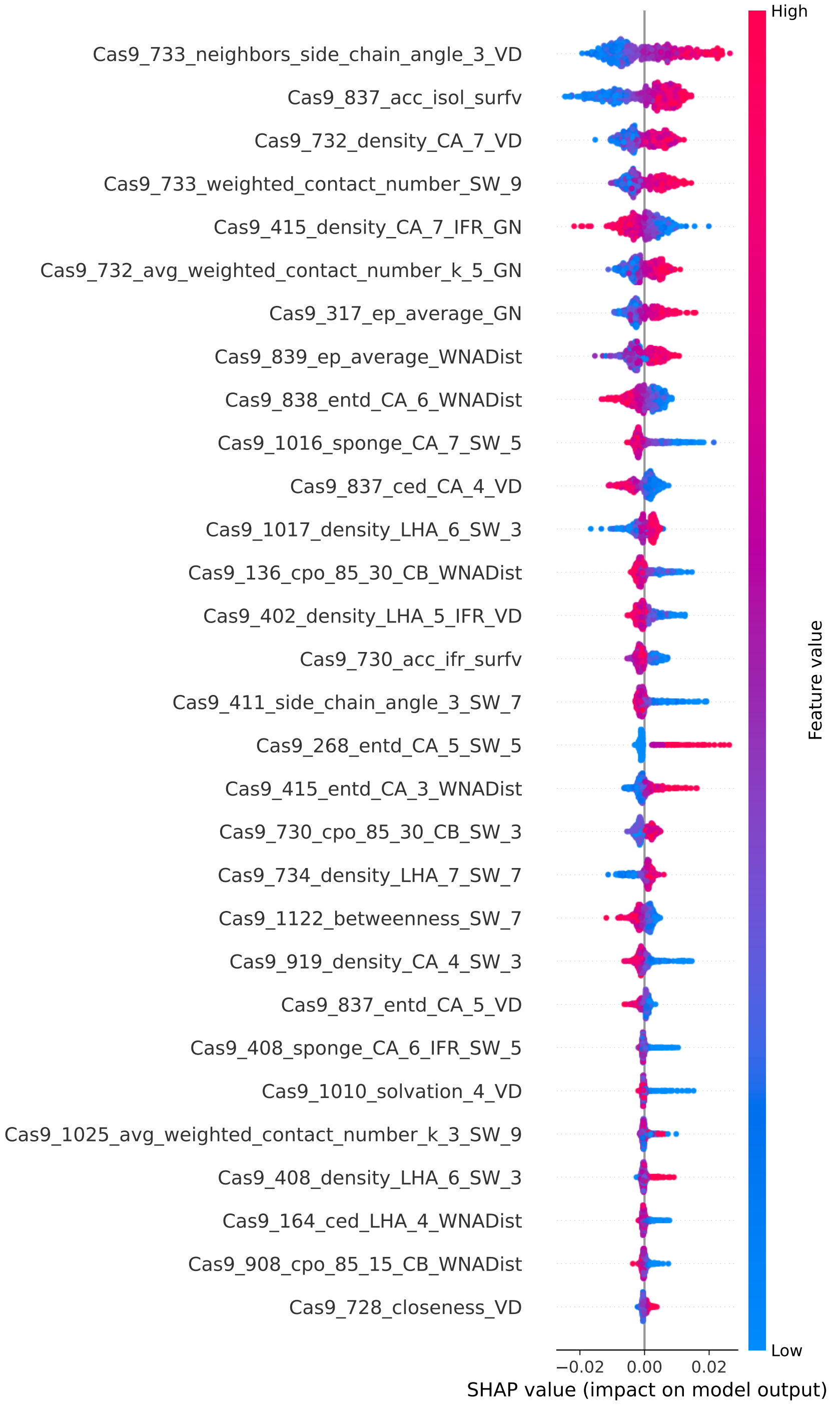

Figure S2: SHAP summary plot for the 30 input features in STING_CRISPR for all 672 PDB snapshots.

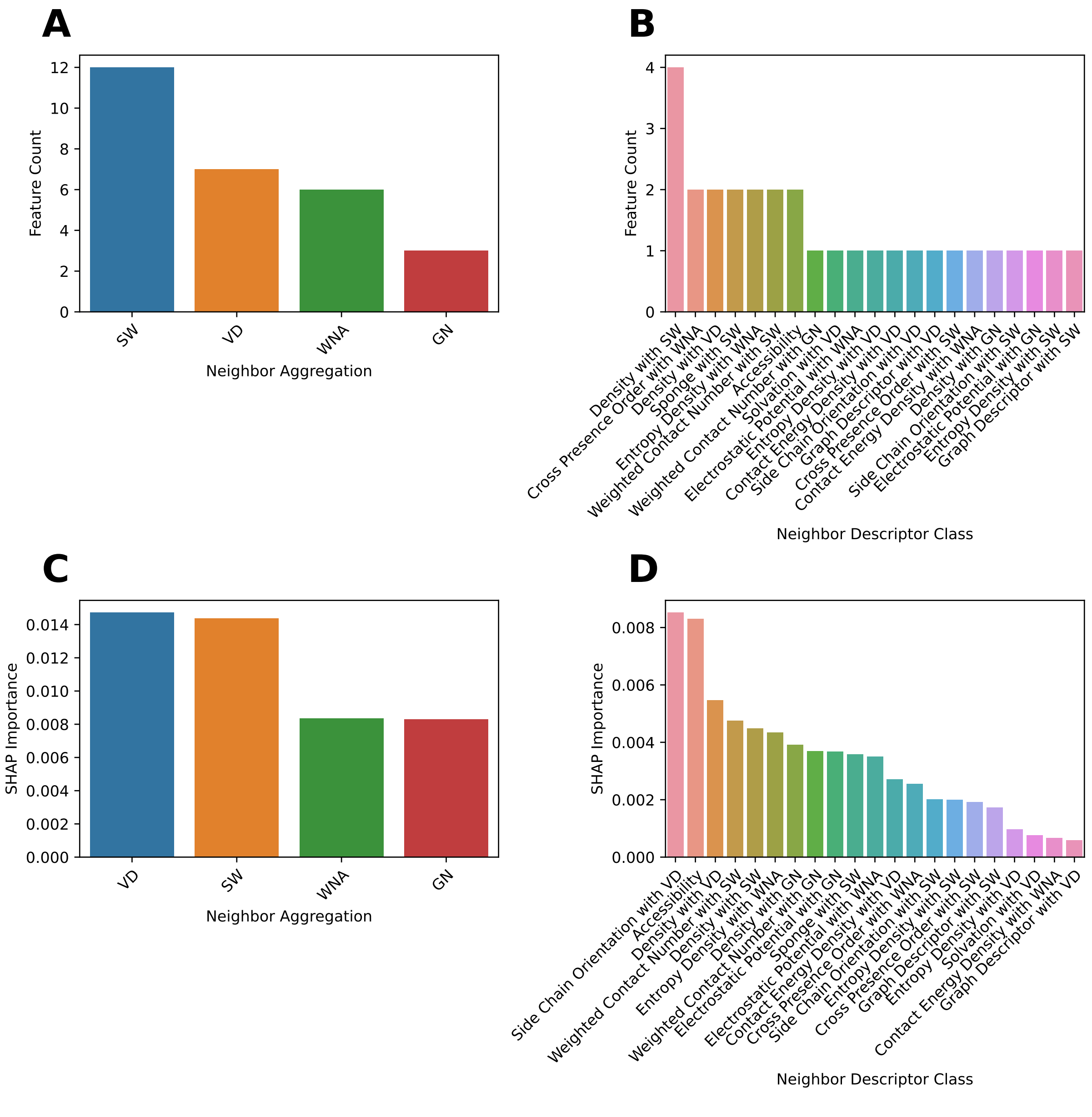

Figure S3: (A, B) The number of features in STING_CRISPR present for each neighbor aggregation method (A) and descriptor class (B), sorted by decreasing feature counts. (Bottom) SHAP importance of neighbor aggregation methods (C) and descriptor classes (D) in STING_CRISPR, sorted by descending SHAP importance.

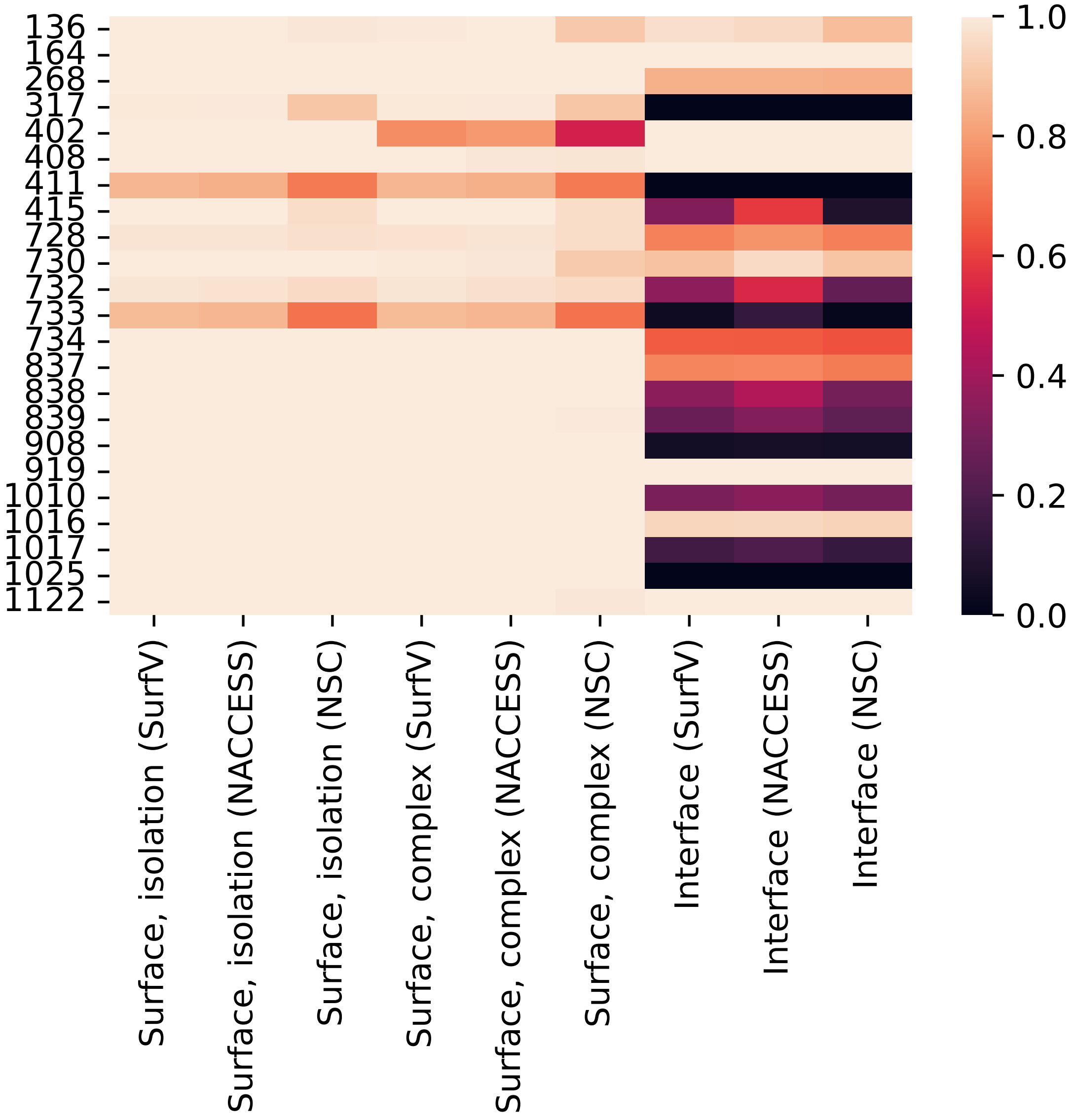

Figure S4: Fraction of the 672 snapshots where a residue in STING_CRISPR is a surface residue in isolation, a surface residue in complex, or is on the interface when accessibility is defined by either SurfV (*1*), NACCESS (*3*) or NSC (*4*).

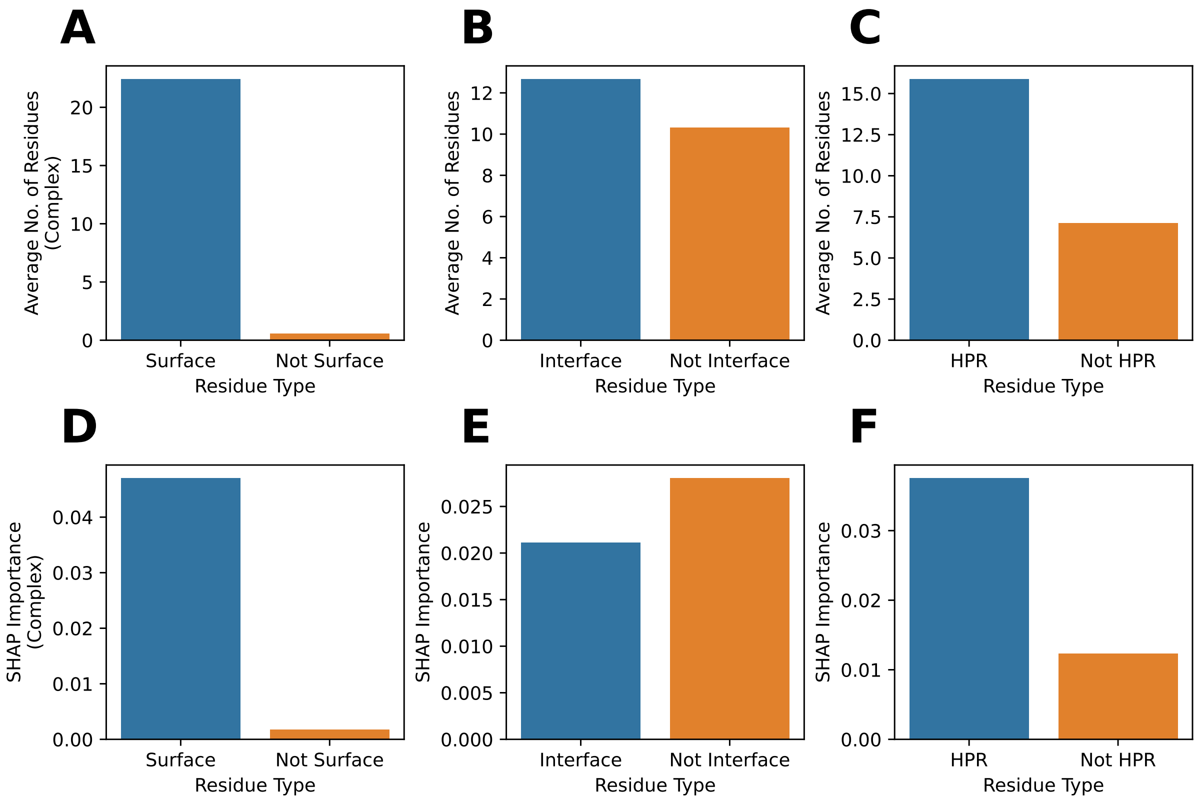

Figure S5: Average number of residues (top) and SHAP importances (bottom) of surface vs. non-surface residues in complex (left), interface vs. non-interface residues (middle), and heteroduplex-proximal residues vs. non-heteroduplex-proximal residues (right). Plots A and C use SurfV (*1*) to determine whether a residue is on the complex's surface or the interface. Results similar plots A and C are obtained when using other tools (NACCESS (*3*) and NSC (*4*)) for measuring solvent accessible area, and/or when categorizing by surface vs. non-surface residues in isolation.

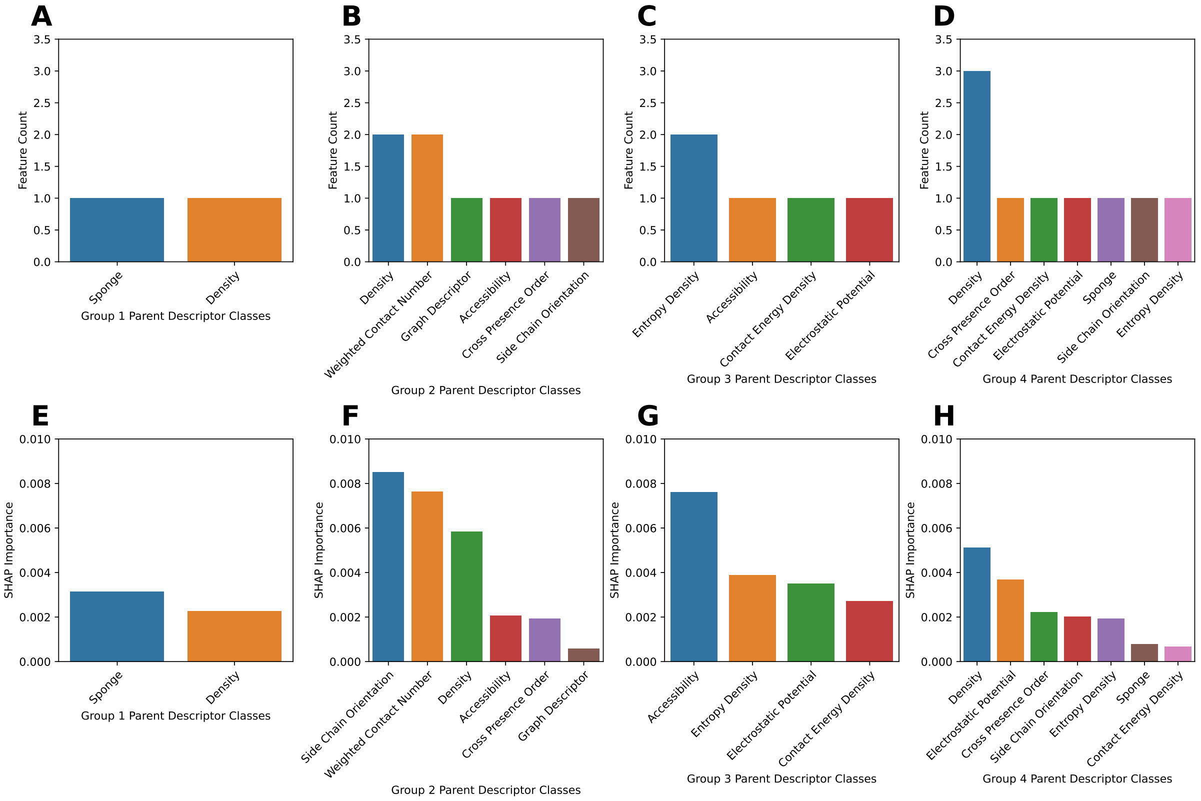

Figure S6: Analysis of 4 residue groups (group 1: 1016, 1017; group 2: 728, 730, 732-734; group 3: 837-839; group 4: 136, 164, 317, 402, 408, 411, 415) and other residues (908, 919, 268, 1122, 1010 and 1025) identified by STING_CRISPR. (Top) Feature counts (left) and SHAP importance (right) of the 4 residue groups and other residues. (Middle) Feature counts of parent descriptor classes for the 4 residue groups. (Bottom) SHAP importance of parent descriptor classes for the 4 residue groups.

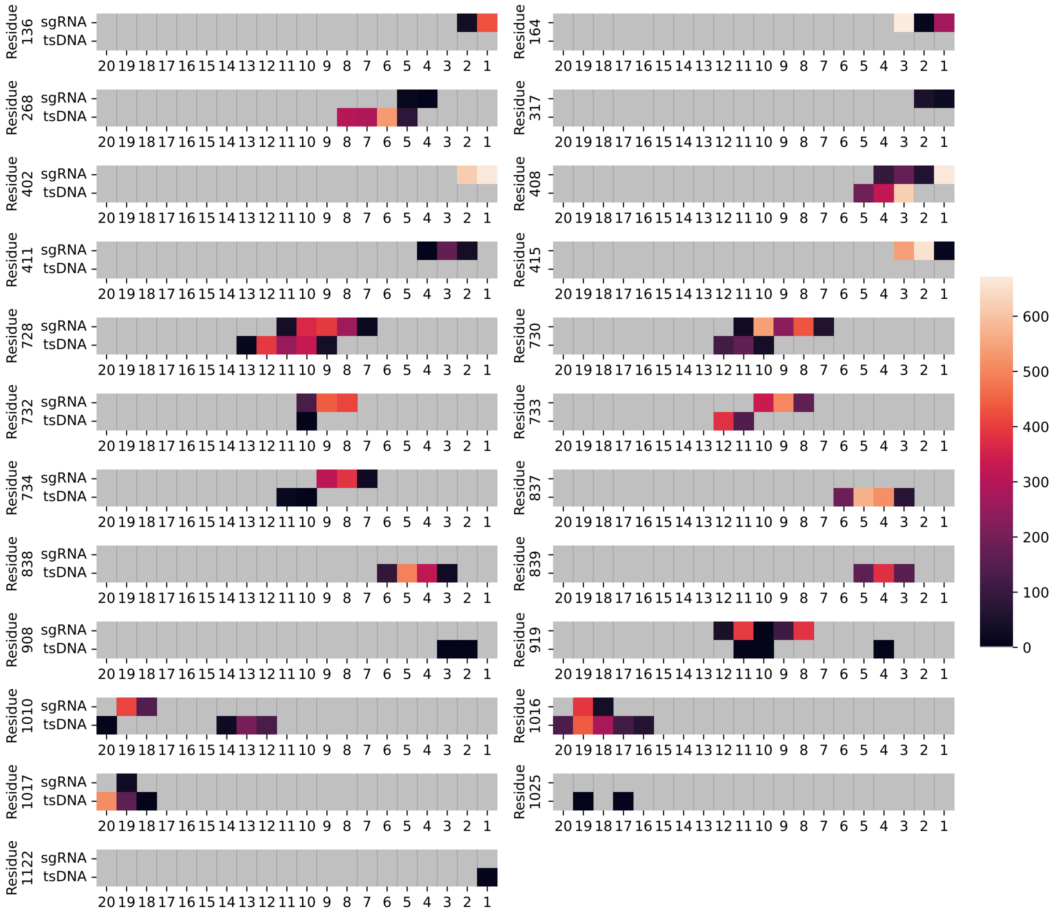

Figure S7: Number of PDB snapshots where a specific amino acid residue has its α-carbon atom $3-7$Å away from a specified sgRNA or tsDNA nucleotide's C4' atom, for the 20 CRISPR-Cas9 residues in STING_CRISPR. The maximum count for a given heatmap cell is 672. Grey cells indicate a count of zero.

1.6 sgRNA-target DNA strand heteroduplex stability

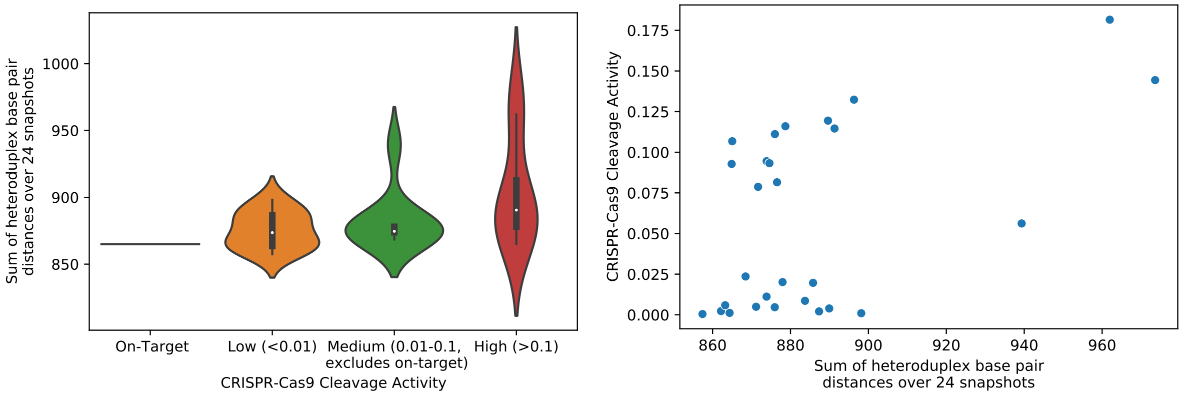

Figure S8: (Left) Sum of the 19 PAM-proximal base pair distances in the sgRNA-tsDNA heteroduplex for the 28 guide-target interfaces, categorized by activity level. On-target refers to the MD trajectory with no base pair mismatches in the heteroduplex. Low, medium and high refer to MD trajectories with CRISPR-Cas9 off-target cleavage activity < 0.01, $0.01 - 0.1$ (excluding on-target interface), and > 0.1, respectively. (Right) Scatter plot of the sum of the 19 heteroduplex base pair distances in the sgRNA-tsDNA heteroduplex versus CRISPR-Cas9 off-target cleavage activity levels, with Spearman and Pearson correlations 0.418 and 0.503, respectively.

1.7 Holding out trajectories as test sets

Test sets for each fold of the five-fold cross validation was constructed by binning snapshots associated with the trajectory with the nth lowest cleavage activity in to the test partition of fold $n \mod 5$, and into the training partition in the other folds. We then used these train-test splits to train linear, ridge, XGBoost, extra trees, and LightGBM regression models, all using default parameters. After model training, we compared the distribution of test squared errors between trained LightGBM models in each of the 5 folds and STING-CRISPR on the last four snapshots of the 5-fold test trajectories.

References

1. Sridharan, S., Nicholls, A., and Honig, B. (1992) A new vertex algorithm to calculate solvent accessible surface-areas. In *Faseb Journal* FEDERATION AMER SOC EXP BIOL

9650 ROCKVILLE PIKE, BETHESDA, MD 20814-3998 Vol. 6, pp. A174–A174.

2. Tsodikov, O. V., Record Jr, M. T., and Sergeev, Y. V. (2002) Novel computer program for fast exact calculation of accessible and molecular surface areas and average surface curvature. *Journal of computational chemistry,* **23**(6), 600–609.

3. Hubbard, S. J., Thornton, J. M., et al. Naccess. `http://www.bioinf.manchester.ac.uk/naccess/` (1993) Last accessed June 4, 2024.

4. Shrake, A. and Rupley, J. A. (1973) Environment and exposure to solvent of protein atoms. Lysozyme and insulin. *Journal of molecular biology,* **79**(2), 351–371.

5. Mancini, A. L., Higa, R. H., Oliveira, A., Dominiquini, F., Kuser, P. R., Yamagishi, M. E., Togawa, R. C., and Neshich, G. (Sep, 2004) STING Contacts: a web-based application for identification and analysis of amino acid contacts within protein structure and across protein interfaces. *Bioinformatics,* **20**(13), 2145–2147.

6. Honig, B. and Nicholls, A. (1995) Classical Electrostatics in Biology and Chemistry. *Science,* **268**(5214), 1144–1149.

7. Rocchia, W. and Neshich, G. (Oct, 2007) Electrostatic potential calculation for biomolecules–creating a database of pre-calculated values reported on a per residue basis for all PDB protein structures. *Genet Mol Res,* **6**(4), 923–936.

8. Sander, C. and Schneider, R. (1991) Database of homology-derived protein structures and the structural meaning of sequence alignment. *Proteins: Structure, Function, and Bioinformatics,* **9**(1), 56–68.

9. Brinda, K. and Vishveshwara, S. (2005) A Network Representation of Protein Structures: Implications for Protein Stability. *Biophysical Journal,* **89**(6), 4159–4170.

10. Dokholyan, N. V., Li, L., Ding, F., and Shakhnovich, E. I. (2002) Topological determinants of protein folding. *Proceedings of the National Academy of Sciences,* **99**(13), 8637–8641.

11. Greene, L. H. and Higman, V. A. (2003) Uncovering Network Systems Within Protein Structures. *Journal of Molecular Biology,* **334**(4), 781–791.

12. Vendruscolo, M., Dokholyan, N. V., Paci, E., and Karplus, M. (Jun, 2002) Small-world view of the amino acids that play a key role in protein folding. *Phys. Rev. E,* **65**, 061910.

13. Agnarsson, G. and Greenlaw, R. (2006) Graph Theory: Modeling, Applications, and Algorithms, Prentice-Hall, Inc., USA.

14. Oehlers, M. and Fabian, B. (2021) Graph Metrics for Network Robustness—A Survey. *Mathematics,* **9**(8), 895.

15. Newman, M. J. (2005) A measure of betweenness centrality based on random walks. *Social Networks,* **27**(1), 39–54.

16. Radzicka, A. and Wolfenden, R. (1988) Comparing the polarities of the amino acids: side-chain distribution coefficients between the vapor phase, cyclohexane, 1-octanol, and neutral aqueous solution. *Biochemistry,* **27**(5), 1664–1670.

17. Kyte, J. and Doolittle, R. F. (1982) A simple method for displaying the hydropathic character of a protein. *Journal of molecular biology,* **157**(1), 105–132.

18. Kabsch, W. and Sander, C. (1983) Dictionary of protein secondary structure: pattern recognition of hydrogen-bonded and geometrical features. *Biopolymers: Original Research on Biomolecules,* **22**(12), 2577–2637.

19. Frishman, D. and Argos, P. (1995) Knowledge-based protein secondary structure assignment. *Proteins: Structure, Function, and Bioinformatics,* **23**(4), 566–579.

20. Chien, Y.-T. and Huang, S.-W. (10, 2012) Accurate Prediction of Protein Catalytic Residues by Side Chain Orientation and Residue Contact Density. *PLOS ONE,* **7**(10), 1–11.

21. Ooi, T., Oobatake, M., Némethy, G., and Scheraga, H. A. (1987) Accessible surface areas as a measure of the thermodynamic parameters of hydration of peptides.. *Proceedings of the National Academy of Sciences,* **84**(10), 3086–3090.

22. Porollo, A. and Meller, J. (2007) Prediction-based fingerprints of protein–protein interactions. *Proteins: Structure, Function, and Bioinformatics,* **66**(3), 630–645.

23. Jones, S. K., Hawkins, J. A., Johnson, N. V., Jung, C., Hu, K., Rybarski, J. R., Chen, J. S., Doudna, J. A., Press, W. H., and Finkelstein, I. J. (01, 2021) Massively parallel kinetic profiling of natural and engineered CRISPR nucleases. *Nat Biotechnol,* **39**(1), 84–93.
